# Sensing of Immature Particles Produced by Dengue Virus Infected Cells Induces an Antiviral Response by Plasmacytoid Dendritic Cells

**DOI:** 10.1371/journal.ppat.1004434

**Published:** 2014-10-23

**Authors:** Elodie Décembre, Sonia Assil, Marine L. B. Hillaire, Wanwisa Dejnirattisai, Juthathip Mongkolsapaya, Gavin R. Screaton, Andrew D. Davidson, Marlène Dreux

**Affiliations:** 1 CIRI, Université de Lyon, Inserm, U1111, Ecole Normale Supérieure de Lyon, Université Lyon 1, CNRS, UMR5308, LabEx Ecofect; Université de Lyon, Lyon, France; 2 Division of Immunology and Inflammation, Department of Medicine, Hammersmith Campus, Imperial College London, London, United Kingdom; 3 Dengue Hemorrhagic Fever Research Unit, Office for Research and Development, Siriraj Hospital, Mahidol University, Bangkok, Thailand; 4 School of Cellular and Molecular Medicine, Faculty of Medical Sciences and Veterinary Sciences, University of Bristol, Bristol, United Kingdom; Icahn School of Medicine at Mount Sinai, United States of America

## Abstract

Dengue virus (DENV) is the leading cause of mosquito-borne viral illness and death in humans. Like many viruses, DENV has evolved potent mechanisms that abolish the antiviral response within infected cells. Nevertheless, several *in vivo* studies have demonstrated a key role of the innate immune response in controlling DENV infection and disease progression. Here, we report that sensing of DENV infected cells by plasmacytoid dendritic cells (pDCs) triggers a robust TLR7-dependent production of IFNα, concomitant with additional antiviral responses, including inflammatory cytokine secretion and pDC maturation. We demonstrate that unlike the efficient cell-free transmission of viral infectivity, pDC activation depends on cell-to-cell contact, a feature observed for various cell types and primary cells infected by DENV, as well as West Nile virus, another member of the *Flavivirus* genus. We show that the sensing of DENV infected cells by pDCs requires viral envelope protein-dependent secretion and transmission of viral RNA. Consistently with the cell-to-cell sensing-dependent pDC activation, we found that DENV structural components are clustered at the interface between pDCs and infected cells. The actin cytoskeleton is pivotal for both this clustering at the contacts and pDC activation, suggesting that this structural network likely contributes to the transmission of viral components to the pDCs. Due to an evolutionarily conserved suboptimal cleavage of the precursor membrane protein (prM), DENV infected cells release uncleaved prM containing-immature particles, which are deficient for membrane fusion function. We demonstrate that cells releasing immature particles trigger pDC IFN response more potently than cells producing fusion-competent mature virus. Altogether, our results imply that immature particles, as a carrier to endolysosome-localized TLR7 sensor, may contribute to regulate the progression of dengue disease by eliciting a strong innate response.

## Author Summary

Viral recognition by the host often triggers an antiviral state, which suppresses viral spread and imparts adaptive immunity. Like many viruses, dengue virus (DENV) defeats the host-sensing pathway within infected cells. However, *in vivo* studies have demonstrated a key role of innate immunity in controlling DENV infection. Here we report that sensing of DENV-infected cells by non-permissive innate immune cells, the plasmacytoid dendritic cells (pDCs), triggers a cell-contact- and TLR7-dependent activation of a strong antiviral IFN response. This cell-to-cell sensing involves transmission of viral elements that are clustered at the interface between pDCs and infected cells and is regulated by the actin network. Importantly, we revealed that uncleaved prM surface protein-containing immature particles play a key function in stimulating the innate immune response. These non-infectious immature particles are released by infected cells as a consequence of a suboptimal cleavage site, which is an evolutionarily conserved viral feature that likely favors the export of infectious virus by prevention of premature membrane fusion in the secretory pathway. Therefore our results highlight a conceptually novel trade-off between efficient infectious virus release and the production of IFN-inducing particles. This concept may have broad importance for the many viruses that, like DENV, can disable the pathogen-sensing machinery within infected cells and can release uncleaved glycoprotein-containing non-infectious particles.

## Introduction

The innate immune system acts as the first line of defense for the sensing of viral infection. This involves rapid recognition of pathogen-associated molecular patterns (PAMPs), including viral nucleic acids, by pattern recognition receptors (PRRs). This recognition results in an antiviral response characterized by the production of type I interferons (IFNs) and expression of IFN-stimulated genes (ISGs). This response suppresses viral spread by blocking the viral life cycle at multiple levels and also mediates immunomodulatory effects in surrounding tissues that impart the onset of the adaptive immune response [1]. The PRR can be cytoplasmic, e.g., retinoic inducible gene-I (RIG-I)-like receptors (RLRs) and NOD-like receptors (NLRs), or endosomal, e.g., Toll-like receptors (TLRs) [1]. Thus, depending on their intracellular localization, virus-induced innate immune signaling typically occurs within cells that are either productively infected or that have internalized viral particles [1], [2].

Recent studies illustrated the existence of alternative host sensing strategies by bystander plasmacytoid dendritic cells (pDCs), which recognize infected cells [3], [4], [5], [6], [7]. pDCs are immune cells known to function as sentinels of viral infection and are a major type I IFN-producing cell type *in vivo* [8], [9]. Using hepatitis C virus (HCV) as a model, we recently demonstrated that HCV infected cells can selectively package immunostimulatory viral RNA within exosomes that deliver their RNA cargo to pDCs, which, in turn, produce IFNα [3]. Exosomes also permit transfer to pDCs of distinct immunostimulatory viral RNAs, such as those of the negative strand lymphocytic choriomeningitis virus (LCMV) [4]. This sensing pathway is thought to assure recognition of infected cells and hence protects the host against viruses that defeat the pathogen-sensing machinery within the cells they infect.

Virtually all viruses have evolved strategies that preclude antiviral signaling in the cell they infect [10]. For example, dengue virus (DENV) has evolved several evasion strategies that prevent IFN and ISG expression within infected cells [11]. Notably, the DENV NS2B-3 protease complex, by cleavage and degradation of an adapter of the cytoplasmic sensor-mediated signaling (STING, also called MITA) and by preventing phosphorylation and nuclear translocation of the downstream transcriptional factor, IFN regulatory 3 (IRF3), inhibits type I IFN production in DENV infected cells [12], [13], [14], [15]. Despite these potent inhibitory mechanisms, expression of antiviral and inflammatory molecules is readily detected in DENV infected humans [16], [17]. Their levels play a pivotal role in DENV infection clearance and pathogenicity [16], [18], [19], thus highlighting the importance of elucidating the host sensing mechanisms leading to the IFN response during DENV infection.

Here, we showed that pDCs are robust IFNα producer cells in response to DENV infected cells. In addition, we demonstrated that cell-to-cell contact- and TLR7-dependent pDC responsiveness leads to an antiviral state, inflammatory cytokine production as well as expression of co-stimulatory molecules by pDCs. Newly formed particles of DENV, like many viruses, undergo maturation by cleavage of the virus envelope protein, premembrane (prM), in the secretory pathway that renders the virus infectious [20]. Yet, the prM cleavage site is suboptimal, leading to the secretion of about 30–40% immature, prM-bearing particles [21], [22], [23], [24], [25], [26]. This evolutionarily conserved suboptimal site may be critical for the export of the infectious viral particles and/or may also positively contribute to viral infection by usurpation of humoral immune response, because anti-prM antibodies facilitate efficient binding and cell entry of prM-containing immature particles into Fc-receptor-expressing cells, a process called antibody dependent enhancement (ADE) [21], [22], [23], [27], [28]. Here, we report a previously unsuspected function of immature particles in innate immunity. Although the immature particles are not infectious, they are fully competent to trigger a robust type I IFN response by contacting non-permissive pDCs. Our results highlight the trade-off between efficient secretion of infectious viral particles and the production of a large amount of IFN-inducing immature particles.

## Results

### IFNα is robustly produced by pDCs in contact with DENV infected cells

**Figure 1:**
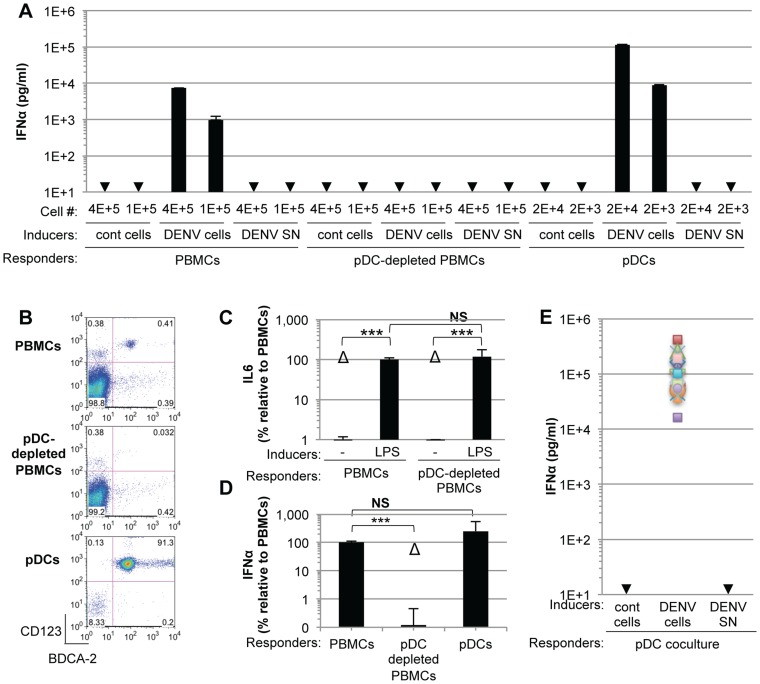
pDCs robustly produce IFNα in response to DENV infected cells. (**A**) Quantification of IFNα in the supernatants of PBMCs, pDC-depleted PBMCs and isolated pDCs (Responders) co-cultured with DENV infected Huh7.5.1 cells (DENV cells) or treated with 100 µl of supernatants from the latter (DENV SN), as indicated (Inducers). Viral titers of DENV SN ≈2.5×10^6^ foci forming units (ffu)/ml. pDC depletion/enrichment was performed using an anti-BDCA-4 antibody. Cell #; number of co-cultured responder cells. Cont cells; uninfected Huh-7.5.1 cells. Arrows indicate results below the detection threshold of the IFNα ELISA (*i.e.*, 12.5 pg/ml). Results are representative of 4 independent experiments. Error bars represent the means ± SD. (**B**) Representative FACS analysis of pDC depletion and isolation from PBMCs using the pDC selective markers, CD123 and BDCA-2. (**C**) Quantification of IL6 in the supernatants of PBMCs and pDC-depleted PBMCs triggered by LPS (10 µg/mL for 20 hours). Results are expressed as percentage relative to LPS-treated PBMCs. 3 independent experiments in triplicate (error bars, means ± SD), paired Student's t test, ***p<0.0001, NS p>0.1, Δ; indicates separated group by ANOVA. (**D**) Total PBMC population containing a number of pDCs equivalent to the purified pDCs as determined by FACS analysis as described in (B) were co-cultured with DENV infected cells. IFNα productions were thus compared for equal numbers of pDCs. PBMCs and pDC-depleted PBMCs were compared using equal cell numbers. The results are expressed as IFNα levels relative to the co-culture with PBMCs, set at 100, 3 independent experiments in triplicate, paired Student's t test, ***p<0.0001, NS p>0.05, Δ; indicates separated group by ANOVA (error bars, means ± SD). (**E**) Quantification of IFNα secretion by pDCs isolated from the blood of a healthy donor cohort (n = 20) co-cultured with infected cells or treated with their supernatant. IFNα levels in the supernatants of pDCs co-cultured with uninfected (cont) cells or with DENV SN were all below the detection threshold (*i.e.*, 12.5 pg/ml).

To investigate the mechanisms regulating the IFN response against DENV infection, primary human peripheral blood mononuclear cells (PBMCs) from healthy donors were exposed to supernatants containing DENV virions or DENV infected cells. We found that PBMCs specifically responded to co-cultivation with DENV infected cells but not to uninfected Huh7.5.1 cells, by a robust secretion of IFNα (Figure 1A). In sharp contrast, supernatants from the DENV infected cells failed to trigger IFNα secretion by PBMCs (Figure 1A). Plasmacytoid dendritic cells (pDCs), which represent a rare PBMC population, *i.e.* 0.41% of PBMCs (Figure 1B, upper panel), are known to produce IFNα [9]. Antibody-mediated pDC depletion from PBMCs (Figure 1B, middle panel) abolished IFNα secretion in response to co-culture with DENV infected cells (Figure 1A). Similar results were also obtained using DENV infected BHK-21 cells (Figures S1A and S1B). To rule out potential non-specific effects of the depletion procedure on innate cell responsiveness, we verified that IL-6 production triggered by lipopolysaccharide (LPS) exposure was maintained after pDC depletion (Figures 1C and S1C). Consistent with the depletion results, the isolated pDC population (Figure 1B, lower panel) potently produced IFNα in response to co-culture with DENV infected cells, but not in the presence of their supernatants (Figure 1A). A very limited number of pDCs (*i.e.*, 2,000 pDCs) was sufficient to produce a robust secretion of IFNα (Figure 1A). Similar levels of IFNα production were detected after co-culture of infected cells with isolated pDCs as compared to total PBMCs, which contained a similar number of pDCs (Figure 1D), further suggesting that pDCs are the main IFNα producer cells among PBMCs. We showed that the cells productively infected with DENV did not produce IFNα themselves (Figure S2A). The pDC IFNα response increased as the duration of infection and, thus the replication levels, prior to co-culture increased (Figure S2A). Remarkably, similar levels of IFNα secretion were reproducibly obtained with pDCs isolated from the blood of a cohort of 20 healthy donors (Figure 1E). Together these results suggest that pDCs represent the main cell type in PBMC populations that produce IFN in response to co-cultivation with DENV infected cells and that this response was not induced by the addition of cell-free supernatants containing virus.

**Figure 2:**
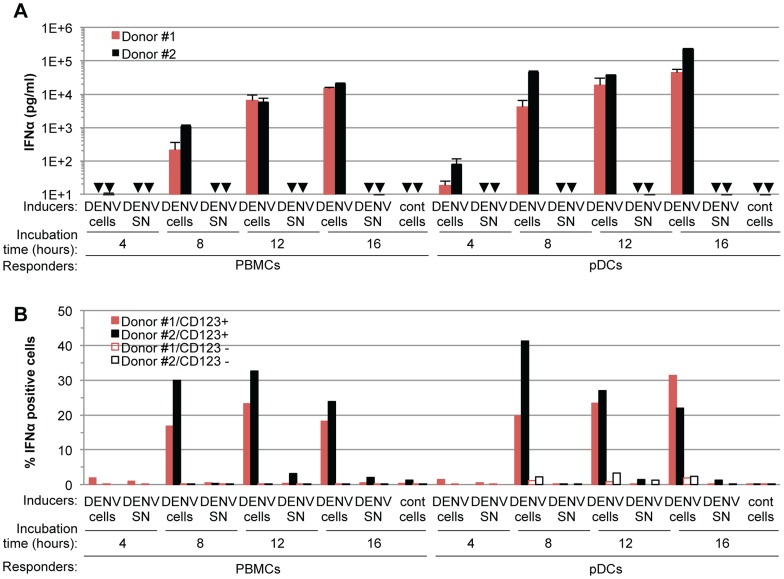
Time course analysis of pDC IFNα production triggered by DENV infected cells. (**A**) Quantification of IFNα in the supernatants of PBMCs and pDCs isolated from two different donors (#1 and #2, as indicated) and co-cultured with DENV infected Huh7.5.1 cells (DENV cells) or treated with 100 µl of supernatant from the latter cells (DENV SN, viral titers ≈3×10^6^ and 1×10^6^ ffu/ml for experiments with Donor #1 and #2, respectively) for the indicated time of incubation. Uninfected Huh-7.5.1 cells are referred to as (cont) cells. Error bars represent the means ± SD, n = 3. Arrows indicate results below the limit of detection of the IFNα ELISA assay (*i.e.* 12.5 pg/ml). (**B**) Quantification of IFNα-positive cells at the indicated incubation times of PBMCs or pDCs isolated from two different donors (#1 and #2, as indicated) and co-cultured with DENV infected Huh7.5.1 cells (DENV cells) or treated with 100 µl of supernatant from the latter cells (DENV SN, viral titers of ≈3×10^6^ ffu/ml). The pDC surface marker CD123 and intracellular IFNα staining were assessed by FACS analysis. Results are expressed as the percentages of IFNα positive cells in CD123 positive- and negative-populations.

To exclude the possibility that pDCs respond transiently to supernatants containing DENV, we quantified IFNα secretion in time course experiments. IFNα secretion was already detectable as early as 4 hours after co-cultivation of pDCs and DENV infected cells (Figure 2A). IFNα levels concurrently increased over the time course of co-culture of DENV infected cells with either pDCs or PBMCs, and reached levels around 100 ng/mL after 16 hours of co-culture (Figure 2A). In contrast, cell-free supernatants containing DENV did not trigger detectable IFNα production by pDCs or by PBMCs at any of time points analyzed (Figure 2A). IFNα producer cells were markedly enriched in pDCs, characterized as a CD123-positive population, as compared to the CD123-negative population (Figure 2B). For example, 12 hours after co-culture of DENV infected cells with PBMCs, ≈0.05% and ≈25–30% of CD123-negative and –positive cells, respectively, were IFNα positive (Figure 2B). Consistently, the frequencies of IFNα producer cells (*i.e.*, about 30%) among pDCs (*i.e.*, CD123-positive populations) were comparable in co-cultures of DENV infected cells with PBMCs *vs.* isolated pDCs (Figure 2B). Together these results suggested that IFNα is robustly produced only by pDCs that are co-cultured with DENV infected cells.

### Sensing of cells infected by different members of the *Flavivirus* genus by pDCs is not cell-type restricted

**Figure 3:**
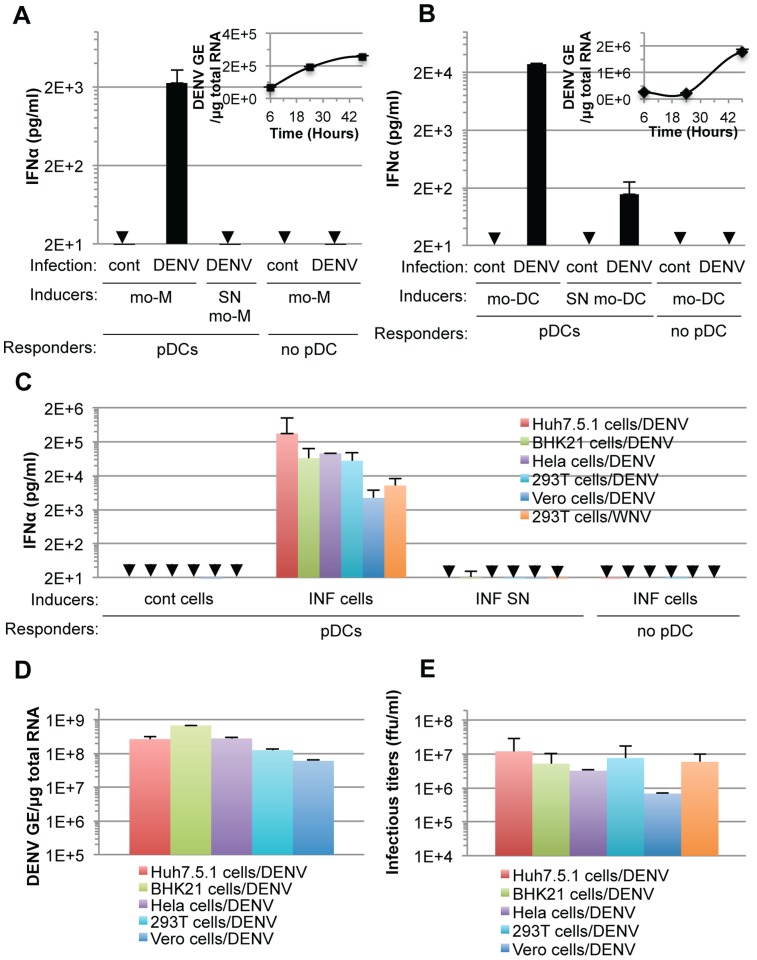
pDC IFNα secretion is triggered by DENV infected primary cells and is not DENV-restricted. (**A–B**) Quantification of IFNα in supernatants of pDCs co-cultured with infected monocyte-derived macrophages (mo-M, DENV) (**A**) or with infected monocyte-derived dendritic cells (mo-DCs, DENV) (**B**) or treated with their supernatant (SN). Error bars represent the means ± SD. Results are representative of 2 independent experiments. Insets, time course analysis of intracellular DENV genome equivalent (GE) levels in mo-M (**A**) and mo-DC (**B**) post-infection with a MOI of 3. Results are expressed as DENV GE/µg total RNA (means ± SD, n = 3). (**C**) Quantification of IFNα in supernatants of pDCs co-cultured with infected cells (INF cells) or treated with 100 µl of supernatant from the latter cells (INF SN). Cont cells, uninfected parental cells. Arrows indicate results below the limit of detection of the IFNα ELISA (*i.e.*, 12.5 pg/ml). Error bars represent the means ± SD (n, independent experiments, in duplicate: Huh7.5.1 cells/DENV, n = 8; BHK-21 cells/DENV, n = 3; Hela cells/DENV, n = 2; 293T cells/DENV, n = 5; Vero cells/DENV, n = 2; 293T cells/WNV, n = 5). Because of the limited numbers of isolated pDCs, each experiment comprises a part of the panel of cell lines, thus DENV infected Huh7.5.1 cells and their supernatants were included in each independent experiment (n = 8) and served as a reference to compare and collate results of independent experiments. Parallel determination of intracellular DENV RNA levels, expressed as DENV GE/µg total RNA (**D**) and infectious virus production, expressed as ffu/ml (**E**).

Next, we showed that co-cultivation of DENV infected primary cells, *i.e.*, monocyte-derived macrophages (mo-M) and monocyte-derived dendritic cells (mo-DC) with pDCs (isolated from the same donor), potently triggered pDC IFNα secretion (Figures 3A and 3B). This stood in stark contrast to the corresponding cell-free supernatants containing virus or the parental uninfected cells did not, or very weakly, induced pDC IFNα production (Figures 3A and 3B). Consistent with the previously reported inhibition of type I IFN production by the DENV NS2B-3 protease in infected cells [12], [13], [14], [15], DENV infected primary cells did not produced detectable levels of IFNα (Figures 3A and 3B).

Additionally, we determined if the production of IFNα by pDCs could be reproduced in response to co-culture with various cell types infected by DENV. Robust secretion of IFNα was triggered when pDCs were co-cultivated with DENV infected cell lines from different origins (*i.e.*, human Huh7.5.1, Hela and 293T cells or non-human BHK-21 and Vero cells), but not by the corresponding supernatants containing virus or the uninfected cells (Figure 3C). DENV infected Vero cells were weaker IFNα inducers (Figure 3C), consistent with lower levels of intracellular DENV RNA (Figure 3D) and infectious viral particle (Figure 3E) produced by these cells, suggesting that pDC IFNα induction is proportional, to some degree, with the level of viral replication.

Remarkably, 293T cells infected by another member of the *Flavivirus* genus, West Nile virus (WNV), but not the corresponding cell-free supernatants containing virus, also triggered robust IFNα production when co-cultured with pDCs (Figure 3C). Similar to the results obtained using co-cultures with DENV infected cells, the pDC IFNα responses increased as the numbers of WNV infected cells increased (Figures S2B and S2C). Together, these results demonstrated that the production of IFNα by pDCs in response to co-culture with DENV infected cells is not cell type specific and that pDCs similarly respond to another member of the *Flavivirus* genus.

### Short range sensing of infected cells by pDCs triggers the IFNα response

**Figure 4:**
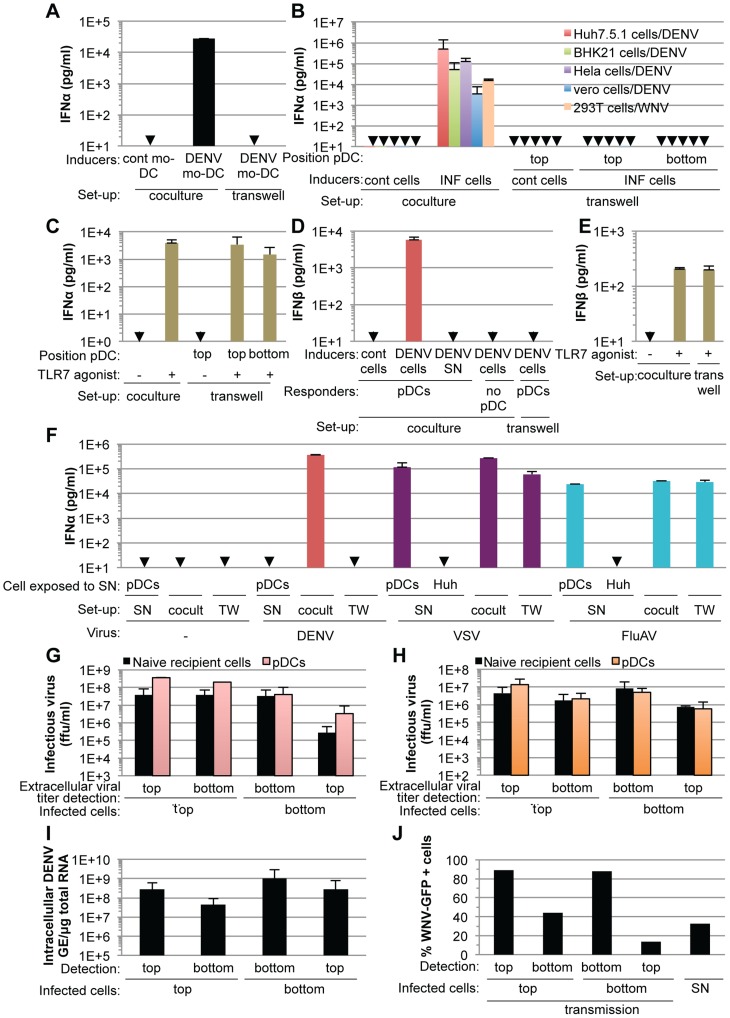
pDC IFNα production triggered by DENV and WNV infected cells is cell-to-cell contact-dependent. (**A**) Quantification of IFNα in supernatants of pDCs co-cultured with DENV infected monocyte-derived dendritic cells (mo-DCs) either seeded together (coculture) or separated by a 0.4 µm transwell membrane (transwell). Results are representative of 2 independent experiments in triplicate (means ± SD). Cont mo-DC; uninfected mo-DC. (**B**) Quantification of IFNα in supernatants of pDCs co-cultured with infected cells (INF cells), set-up as in (A). Positions of the pDCs relative to the transwell membrane are indicated. Error bars represent means ± SD, n; independent experiments in duplicate: BHK-21 cells/DENV, n = 4; Hela cells/DENV, n = 2; Vero cells/DENV, n = 2; 293T cells/WNV, n = 2. Huh7.5.1 cells/DENV were included in each independent experiment (n = 5) as a reference. Cont cells; uninfected parental cells. (**C**) Quantification of IFNα in supernatants of pDCs triggered by a TLR7 agonist (R848, 50 ng/mL), set-up as in (A) (means ± SD, n = 5). (**D–E**) Quantification of IFNβ in supernatants of pDCs co-cultured with DENV infected Huh7.5.1 cells (DENV cells) (**D**) or triggered by TLR7 agonist (**E**), set-up as in (A) (means ± SD, n = 3). (**F**) Quantification of IFNα in supernatants of pDCs co-cultured with cells infected, or not (-) by either DENV, vesicular stomatitis virus (VSV) or influenza A virus (FluAV), set-up as in (A). Cocult; co-culture, TW; transwell membrane set-up. pDCs or naïve cells (indicated as Huh) were exposed to the supernatants of infected cells (SN) (means ± SD, n = 3). (**G–H**) Results of viral titers of DENV (**G**) and WNV (**H**) in supernatants harvested from the two sides (top or bottom) of the transwell membrane (means ± SD, 2 independent experiments in duplicate). Positions of the infected cells in reference to the transwell membrane (top or bottom), which separates them from naïve recipient cells or pDCs, are indicated. (**I–J**) Quantification of intracellular DENV RNA levels (**I**) and percentage of WNV-GFP positive cells (**J**) in infected donor cells and co-cultured naïve recipient cells separated by the transwell membrane (means ± SD, 2 independent experiments in duplicate). Parallel infection of 293T cells with supernatant (SN, MOI = 4) serves as a reference for the expected level of WNV-GFP expression at 20 hours post-inoculation (means ± SD, n = 2).

Cell-free supernatants containing virus from various infected cell types failed to trigger pDC IFNα production, even when added as crude non-filtered supernatants containing virus at concentrations as high as 20 infectious units per pDC (Figure S3), indicating that the transmission of the immunostimulatory signal to pDCs likely requires cell-to-cell contacts. To determine if contacts with DENV infected cells favors pDC sensing, we assessed IFNα production by pDCs cultured in transwell chambers with infected cells. Transwell cultures containing DENV infected monocyte-derived dendritic cells (mo-DCs) and pDCs separated by a 0.4 µm permeable membrane did not result in detectable levels of IFNα production by the pDCs (Figure 4A). Similar results were obtained using DENV infected Huh7.5.1, BHK-21, Hela and Vero cells as well as WNV infected cells (Figure 4B), confirming that this feature is not cell type specific or restricted to DENV. Similarly to IFNα, pDCs robustly produced IFNβ when in contact with DENV infected cells, but not when cells were physically separated by a transwell membrane (Figure 4D). Consistent with these results, IFNβ production by pDCs was not triggered by supernatants from DENV infected cells and DENV infected cells did not themselves release detectable levels of IFNβ (Figure 4D). In control experiments using identical transwell culture settings, an agonist of TLR7, a viral RNA immune sensor [9], triggered the production of both IFNα and IFNβ by the pDCs at levels similar to those obtained in the co-culture setting (Figure 4C and 4E), thus ruling out potential non-specific effects of the experimental setting on pDC responsiveness. In agreement with previous reports [29], [30], vesicular stomatitis virus (VSV) or Influenza virus (FluAV) containing supernatants robustly triggered IFNα production by pDCs (Figure 4F). Consistent with these results, VSV and FluAV infected cells in contact with pDCs (Figure 4F, cocult) or separated by a transwell membrane (Figure 4F, TW), triggered IFNα production at similar levels. This suggested that contact with virus infected cells is not a universally employed mechanism to promote pDC activation by RNA viruses. Next, viral transmission across the transwell-membrane was assessed by quantifying infectious DENV (Figure 4G) and WNV (Figure 4H) on both sides of the membrane that separated infected cells from recipient cells. To evaluate the possible interference of recipient cells on the extracellular infectivity detection, we compared two types of recipient cells, *i.e.*, IFNα response-competent pDCs, which are non-permissive to infection (Figure S4) and permissive cells (Figure 4G and 4H, Naïve recipient cells). As expected from their size, infectious viral particles readily flowed across the 0.4 µm membrane (Figures 4G and 4H), thereby permitting viral transmission from infected cells to naïve cells in the absence of direct contact (Figures 4I and 4J). In sharp contrast, type I IFN production by the pDCs was induced exclusively under conditions where cell-to-cell contact was possible between infected cells and pDCs (Figures 4A, 4B and 4D).

Collectively, these results demonstrated that the exposure of pDCs to the DENV or WNV infected cell milieu either at defined time points (Figure 3A–C) or continuously (Figures 4A, 4B and 4D) failed to trigger a robust IFN response by pDCs, which were responsive to infected cells by cell-to-cell contact and/or in a short-range manner.

### DENV infected cells transmit viral RNA to pDCs and induce a TLR7-mediated antiviral state and pDC maturation

**Figure 5:**
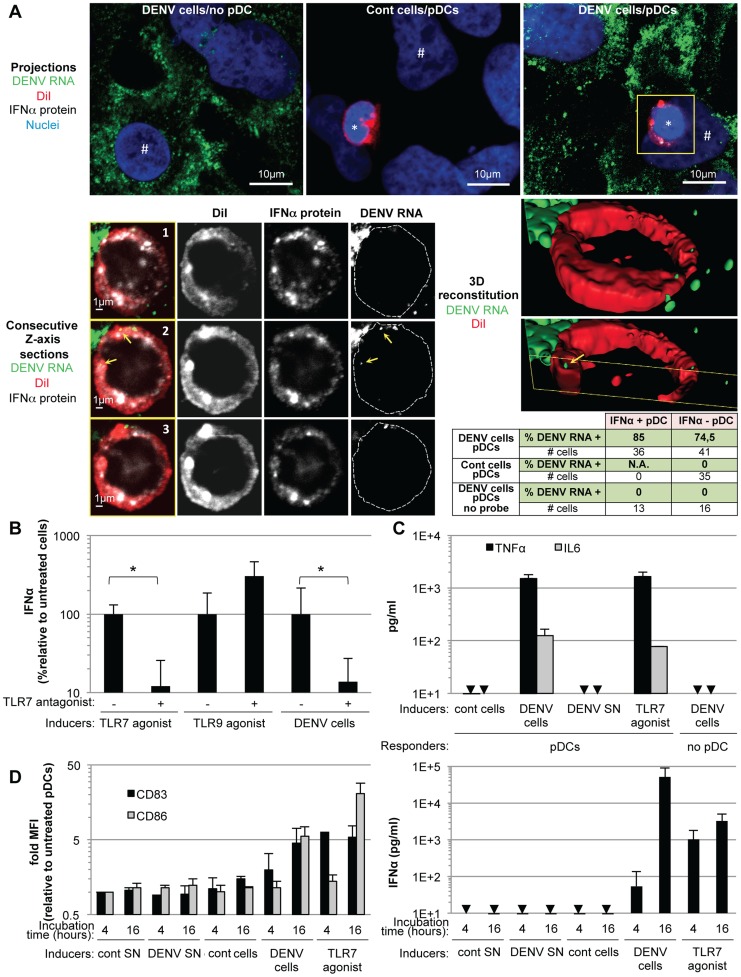
Recognition and pDC antiviral signaling triggered by DENV infected cells. (**A**) DENV RNA is transferred from infected cells to pDCs. Upper panels, projections of confocal microscopy analysis of DENV RNA (green) detected by FISH in DENV infected Huh-7.5.1 cells (DENV cells/no pDC) and in pDCs after 5 hours of co-culture with uninfected cells (cont cells/pDCs) or with DENV infected cells (DENV cells/pDCs), pDC DiI dye (red), IFNα protein (white) and nuclei (blue). Lower panels, consecutive Z-axis sections and 3D reconstitutions with magnification of the yellow-boxed pDC. Cell contours on the DENV RNA panels labeled with dotted lines surrounding DiI staining. Yellow arrows; DENV RNA dots inside pDC, *; pDC and #; Huh7.5.1 cells. Summary table, percentages of IFNα-positive- or negative-pDCs (green colored lines) that contain DENV RNA after co-culture with DENV cells, control cells or DENV cells with FISH procedure performed in absence of DENV probe (no probe), as controls. Similar results were obtained in 3 independent experiments, # cells; numbers of cells included in the analysis. (**B**) Quantification of IFNα in the supernatants of pDCs that were preincubated, or not, with TLR7 antagonist (IRS661, 0.35 µM), as indicated, then co-cultured with DENV infected Huh7.5.1 cells (DENV cells) or stimulated by TLR7 agonist (R848, 50 ng/mL) or TLR9 agonist (ODN2216, 0.1 µM) [1]. Results are expressed as the percentages of IFNα produced in the absence of TLR7 antagonist (means ± SD, n = 4, paired Student's t test: *, p<0.01). (**C**) Quantification of TNFα and IL6 in supernatants of pDCs co-cultured with DENV cells, treated with DENV SN (viral titers ≈2×10^6^ ffu/ml) or TLR7 agonist, set-up as in (B) (means ± SD, n = 2 for IL6, n = 3 for TNFα). Arrows indicate results below the limit of detection (TNFα and IL6, 8 and 25 pg/ml, respectively). (**D**) Left panel, quantification of surface CD83 and CD86 on CD123-gated cells by FACS analysis at the indicated times, set-up as in (B), Right panel, parallel quantification of supernatant IFNα (means ± SD, n = 3).

pDCs typically respond to viral infection *via* endolysosome-localized TLR7- or TLR9 sensors that recognize RNA or DNA viral genomes, respectively [9]. Accordingly, we examined the transmission of DENV RNAs to co-cultured pDCs. The presence of DENV RNA in infected cells and co-cultured pDCs (selectively labeled with DiI, a fluorescent membrane dye) was assessed using a highly sensitive DENV RNA-specific fluorescence *in situ* hybridization (FISH) assay (Figure 5A, upper panels). The analyses were performed after 5 hours of co-culture with DENV infected cells, at which time pDCs already produced IFNα (Figure 2A). DENV RNA (green) was detected as discrete dots inside pDCs (Figure 5A, lower panels). Inspection of consecutive Z-axis sections of co-cultures stained by combined DENV RNA FISH and anti-IFNα immuno-detections revealed that the frequency of DENV RNA-positive pDCs was elevated in both IFNα-positive (*i.e.*, 85%) and IFNα-negative pDCs (*i.e.*, 74.5%) (Figure 5A, summary table). The specificity of these examinations was validated by the absence of DENV RNA-positive pDC when co-cultured with uninfected cells and when the FISH procedure was performed in the absence of the DENV RNA specific probe (Figure 5A, summary table and Figure S5). The presence of DENV RNA in IFNα-negative pDCs may reflect the time required for DENV RNA to trigger pDCs to produce enough IFNα to be detectable, which may not have occurred by 5 hours of co-cultivation. Alternatively, differential DENV RNA localization in intracellular compartments may modulate their recognition by innate sensors, and/or potential subsets of pDCs may be differentially responsive to the DENV RNA stimulus, in accordance with the maximal detection of about 30% IFNα-positive pDCs at plateau (Figure 2B). Only a few DENV RNA dots were detected inside pDCs, suggesting that it is a rare event but sufficient to trigger pDC IFN production. Together, these results indicated that DENV RNA was readily transmitted from DENV infected cells to co-cultured pDCs, supporting the notion that DENV RNA might be recognized by pDC TLR7. Accordingly, a TLR7 antagonist significantly inhibited pDC IFNα production induced by DENV infected cells (Figure 5B). The specificity of this TLR7 antagonist was demonstrated by the inhibition of IFNα production induced by a TLR7 agonist (R848) but not by a TLR9 agonist (ODN2216) (Figure 5B). Collectively, these results suggested that DENV infected cells transfer viral RNA to co-cultured pDCs and trigger TLR7-dependent IFNα production.

Next, to further define the nature of the pDC-mediated antiviral state induced by contact with DENV infected cells, we examined the secretion of the inflammatory cytokines, IL-6 and tumor necrosis factor (TNF)-α, triggered by activation of the transcription factor NF-κβ, known to transduce antiviral signaling downstream of TLR7 [1]. TNF-α is known to play a pivotal role in the vascular leakage syndrome, a hallmark of dengue hemorrhagic fever [18]. Sensing of DENV infected cells, but not their supernatants, specifically triggered pDCs to produce IL-6 and TNF-α at levels comparable to those induced by treatment with a TLR7 agonist (Figure 5C). In addition, ISGs (*i.e.*, MxA and ISG56) were specifically up-regulated in co-cultures of DENV infected cells with pDCs or PBMCs (Figure S6), thus indicating the establishment of an antiviral state.

Finally, we determined if DENV infected cells trigger pDC maturation as assessed by the up-regulation of the CD83 and CD86 markers at the cell surface. DENV infected cells, but not their supernatants, triggered a rapid increase in the surface expression of CD83 on co-cultivated pDCs (*i.e.*, in CD123 marker-gated cells) (Figure 5D, left panel), accompanied by a slightly delayed augmentation of CD86 cell surface expression (Figure 5D, left panel) and by a concomitant increase in IFNα secretion (Figure 5D, right panel). Collectively, these results demonstrated that sensing of DENV infected cells by TLR7, a sensor of single stranded-RNA, triggers IFNα production by pDCs, along with the induction of the inflammatory response, an antiviral state and pDC maturation.

### The IFNα response by pDCs is modulated by DENV glycoproteins

**Figure 6:**
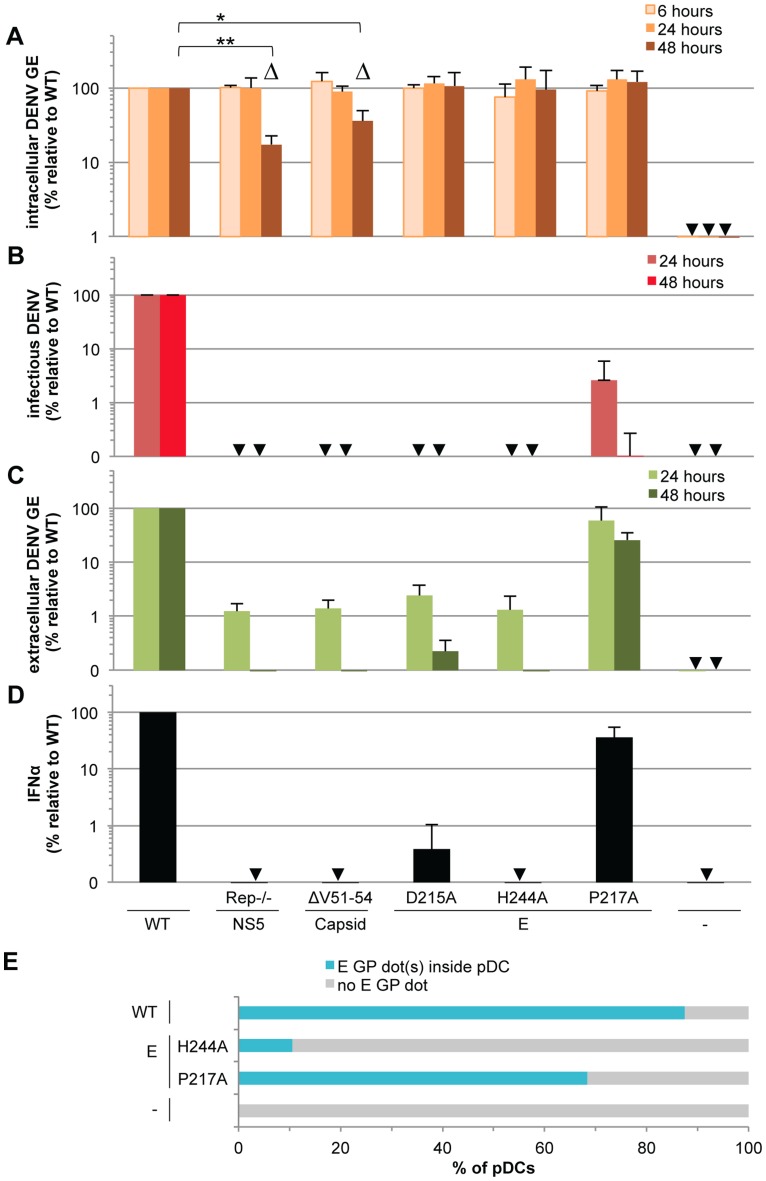
Viral functions required for pDC IFNα production triggered by DENV infected cells. The impact of mutations in NS5 (Rep^−/−^, substitutions G81A, G83A and G85A), capsid (ΔV51–54, deletion V51-to-L54) and E (substitutions D215A, H244A or P217A) on the levels of intracellular DENV RNA (**A**), infectious virus production (**B**), extracellular DENV RNA (**C**) analyzed at the indicated times post-transfection of WT and mutant DENV genomes and quantification of IFNα secretion by pDCs co-cultured with cells harboring the WT and mutant DENV genomes (**D**), control cells (-). GE; genome equivalent. In D, cells harvested at 24 hours post-transfection were co-cultured with pDCs for 20 hours. Results of 3 independent experiments in triplicates are expressed as percentages relative to the WT DENV genome. Error bars represent the means ± SD, paired Student's t test, *p<0.05, **p<0.01. Triangles indicate the mutants (*i.e.*, Rep−/− and ΔV51–54) that belong to a separated group, statistically different from the WT and the other mutants of the panel in regards to the intracellular DENV GE levels at 48 hours post-transfection using a non-parametrical analysis (ANOVA). Arrows indicate results below the limit of detection of RT-qPCR: 20 intracellular DENV GE/µg total RNA and 30 extracellular DENV GE/ml; infectious titers: 20 ffu/ml and IFNα ELISA: 12.5 pg/ml. (**E**) DENV E GPs are transferred from cells harboring the WT and mutant DENV genomes to pDCs in co-cultures for 8 hours. Results are expressed as the percentages of DiI stained pDCs that contain E GP dot(s) detected by immunostaining and analyzed by confocal microscopy. Representative pictures are displayed in Figure S8. Similar results were obtained in 3 independent experiments and ≈20 pDCs surrounded by E GP positive/DiI negative cells were observed per experimental condition.

To define how pDCs sense DENV infected cells, we analyzed the ability of cells harboring recombinant DENV genomes containing mutations specifying phenotypes deficient in various viral functions to trigger IFNα production by co-cultured pDCs. First, we tested cells containing DENV genomes encoding lethal mutations in the methyltransferase domain of the viral NS5 polymerase (*i.e.*, Rep^−/−^) [31]. As expected [31], the triple mutation significantly reduced the intracellular level of DENV RNA at 48 hours post-transfection as compared to the wild type (WT) genome (Figure 6A), reflecting a failure to amplify viral RNA (Figure S7A). Consistently, this mutant did not express detectable amounts of intracellular viral proteins (Figure S7B). Despite comparable intracellular viral RNA levels between the DENV WT and Rep^−/−^ mutant genomes at the onset of co-culture *i.e.*, 24 hours post transfection, likely reflecting the input transfected RNA (Figure 6A), Rep^−/−^ DENV mutant genome harboring cells did not trigger IFNα production by co-cultured pDCs (Figure 6D). Similarly, cells harboring DENV genomes encoding a four amino acid deletion in the capsid (*i.e.*, amino acids V51-to-L54), that significantly compromised both viral RNA replication (Figures 6A and S7A) and viral protein expression (Figure S7B), failed to induce IFNα production by co-cultured pDCs (Figure 6D). Together these results indicated that the pDC IFNα response requires active viral replication in neighboring DENV infected cells.

Next, to address the requirement of viral genome release for pDC activation, we tested the effects of co-culture with cells harboring DENV genomes encoding point mutations in the envelope (E) glycoprotein, *i.e.*, the substitutions D215A, H244A or P217A, known to inhibit infectious viral production [32], [33]. Consistent with previous reports [32], [33], the E glycoprotein mutations did not impair intracellular levels of either viral RNAs or proteins (Figures 6A, S7A and S7B), but they all greatly compromised the production of infectious particles (Figure 6B). Both the D215A and H244A mutations abrogated the release of viral RNA and structural proteins and the pDC IFNα response (Figures 6C–D and S7C). Conversely, cells harboring DENV genomes encoding the P217A mutation triggered the IFNα response by pDCs (*i.e.*, ≈36% relative to WT) (Figure 6D) at various inducer cell concentrations (Figure S7D) and in proportion to the release of extracellular DENV RNA (*i.e.*, ≈60% and 26% relative to WT at 24 and 48 hours post-transfection, respectively) (Figure 6C) and viral structural proteins (Figure S7C). Remarkably, the production of infectious virus (Figure 6B) was severely and disproportionally inhibited by the P217A mutation (*i.e.*, 40-to-1,000 fold-reduction at 24-to-48 hours post-transfection) as compared to the modest inhibition of the IFNα response by pDCs (*i.e.*, ≈2.5 fold-reduction of IFNα response in the same time period) (Figure 6D and S7D). These results suggested that infectious virus production is not required and/or is not rate-limiting for pDC activation. Consistently, pDCs were not permissive to DENV infection (Figure S4A), this latter observation is in line with the previous demonstration of pDCs as refractory to infection by other viruses [30], [34], [35].

Altogether, these results suggested that glycoprotein-dependent release of non-infectious viral components by DENV infected cells might trigger the IFNα response by contacting pDCs.

### DENV envelope protein-dependent transfer and activation of IFNα response by pDCs

To determine whether DENV surface proteins mediate the transmission of viral components to pDCs, we first assessed whether, similarly to DENV RNA (Figure 5A), the DENV envelope proteins are transmitted into the pDCs, by inspection of consecutive Z-axis sections of DiD-labeled pDCs in co-culture with cells harboring the WT and DENV genomes encoding E protein mutations (Figure S8). Similar to DENV genome, we observed the E glycoproteins (E GP) in dot-like structures inside the pDCs. The frequencies of E GP dot-positive pDCs were elevated when in the co-cultures with either cells harboring the WT genome (*i.e.*, around 90%) or the P217A mutation (*i.e.*, above 65%) (Figures 6E and S8), which was in proportion to the release of extracellular DENV RNA (*i.e.*, 60% relative to WT particles at 24 hours post-transfection) (Figures 6C). In contrast, cells harboring the DENV genome encoding the H244A mutation in E, which do not release viral particles and fail to trigger the IFN response by pDCs (Figures 6B, 6C, 6D, S7C and S7D), demonstrated little to no transmission of the E GP into the pDCs (Figures 6E and S8). Because the intracellular levels of viral components (*i.e.*, viral RNA, E and capsid proteins) were equivalent for cells harboring DENV genomes encoding the H244A point mutant, as compared to WT genome (Figures 6A and S7B), the results suggested that pDC IFNα production is activated by the glycoprotein-mediated transmission of viral components from DENV infected cells into contacting pDCs.

Next, we tested the impact of expressing the DENV surface proteins alone (Figure S9A) on pDC IFN induction. Expression of the envelope proteins alone is known to result, in absence of nucleocapsid, in the release of viral envelope containing-membrane vesicles, the sub-viral particles (SVPs) (Figure S9B) [36]. Although the glycoproteins were readily transmitted from cells expressing only the DENV surface proteins to the co-cultured pDCs (Figure S9D–F), IFNα production was not triggered (Figure S9C). These observations are in agreement with the transmission of DENV RNA to pDCs and activation by the TLR7 RNA sensor (Figure 5A and 5B).

To corroborate these results, we determined whether pDC activation by contact with DENV infected cells requires an internalization-dependent mechanism by testing inhibitors of dynamin (Dynasore) [37], of clathrin-mediated endocytosis (Chlorpromazine [38]) and of macropinocytosis (Gö6983-PKC inhibitor [39], [40]). Inhibitors of both dynamin and clathrin-mediated endocytosis, but not macropinocytosis, abrogated pDC IFNα production triggered by DENV infected cells (Figure S10A), without any effect on the ongoing DENV replication and viral production (Figures S10B–C). In addition, these inhibitors did not markedly impair pDC IFNα production induced by a TLR7 agonist (Figure S10A), a cell-permeable imidazoquinoline, which passively diffuses inside the pDCs [41], thus ruling out potential side-effect downstream of TLR7 recognition. These results, in agreement with the requirement of the endolysosome localized-sensor, TLR7 (Figure 5B), suggested that pDC IFNα production triggered by DENV infected cells requires glycoprotein-mediated secretion of non-infectious viral components, which are subsequently internalized by co-cultured pDCs. These results demonstrated that pDC activation triggered by DENV infected cells is distinct from that induced by cells infected by other viruses, such as HCV, LCMV and classical swine fever virus (CSFV), which does not require viral structural protein expression [4], [5], [7].

### Disruption of cell contact and DENV surface protein clustering by cytoskeleton inhibitors abrogates the pDC IFNα response

**Figure 7:**
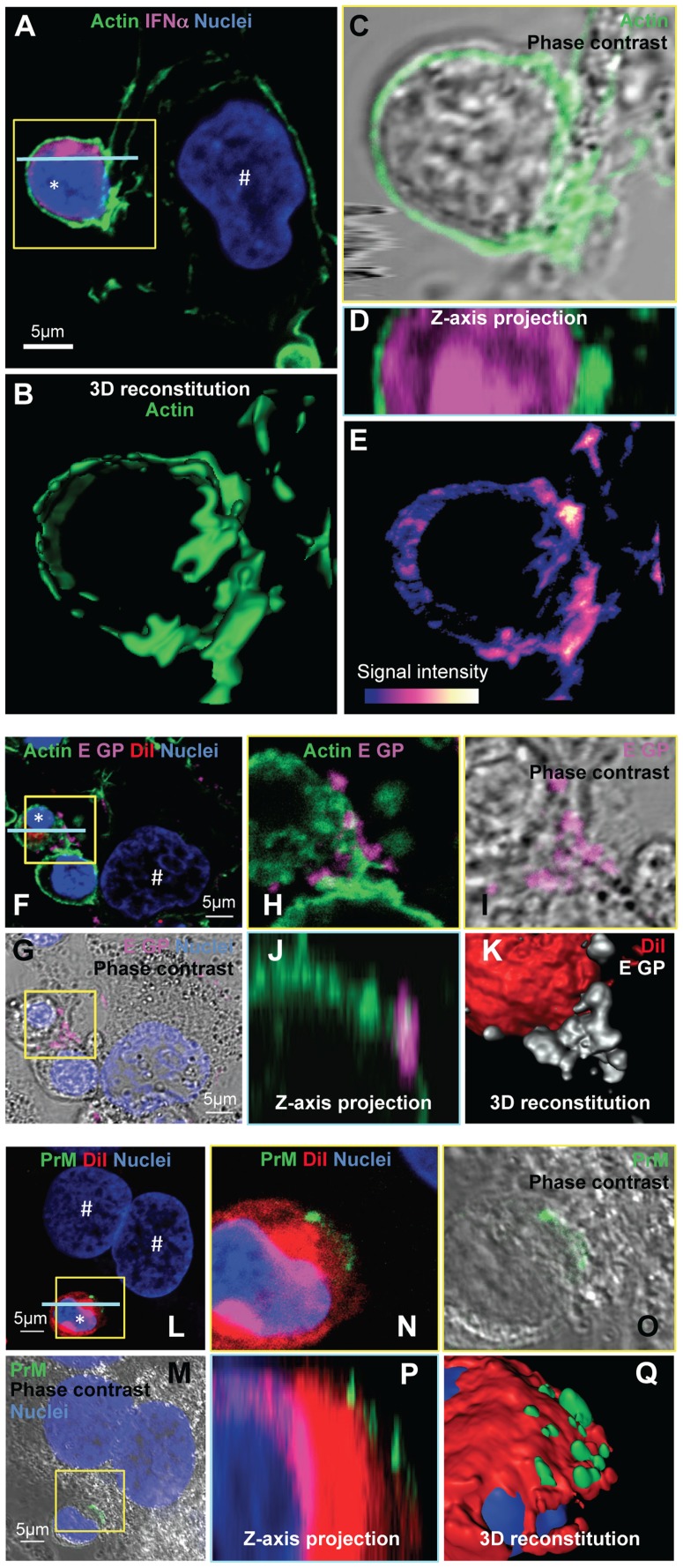
Accumulation of actin network and clustering of DENV surface protein at the cell contacts. (**A–E**) Imaging of the actin network in co-cultures of pDCs and DENV infected Huh7.5.1 cells (8 hour-incubation). (**A**) Confocal microscopy analysis of actin stained with CF488A-conjugated phalloidin (green), IFNα detected by immunostaining (purple) and nuclei stained with Hoechst (blue). (**B–E**) Magnification of yellow-boxed pDC/DENV infected cell contact, shown in (A). (**B**) 3D reconstitution. (**C**) Confocal microscopy analysis of actin projected on the phase contrast imaging. (**D**) Z-axis projection corresponding to the blue line, shown in (A). (**E**) Color-coded image to represent fluorescence intensity with gradient indicating the intensity from weak (blue) to strong signal (white). Similar results were obtained in 3 independent experiments. Star mark (*): pDCs and hash mark (#): Huh7.5.1 cells. (**F–K**) Imaging of DENV E glycoprotein (E GP) clustering at cell contacts between pDC and DENV infected cells (co-cultures for 8 hours). (**F**) Confocal microscopy analysis of with actin (green), pDCs stained with DiI membrane dye (red), E GP (purple, immunodetection without permeabilization step) and nuclei (blue). (**G**) Confocal microscopy analysis of E GP-nuclei projected on the phase contrast imaging. (**H–K**) Magnification of yellow-boxed cell contacts, shown in (F). (**H**) Actin-E GP detection. (**I**) E GP projected on phase contrast. (**J**) Z-axis projection corresponding to the blue line in (F). (**K**) 3D reconstitution. Similar results were obtained in 3 independent experiments. (**L–Q**) Imaging of DENV prM clustering at cell contacts between pDC and DENV infected cell in co-culture for 8 hours. (**L**) Confocal microscopy analysis of prM (green, immunodetection without permeabilization step), DiI-stained pDC (red) and nuclei (blue). (**M**) Confocal microscopy analysis of prM-nuclei projected on the phase contrast imaging. (**N–Q**) Magnification of yellow-boxed cell contacts, shown in (L). (**O**) prM staining projected on phase contrast. (**P**) Z-axis projection corresponding to the blue line in (L). (**Q**) 3D reconstitution. Results are representative of 3 independent experiments.

Next, we sought to study the regulation by cell contacts of DENV surface protein-dependent transfer and activation of pDCs. First, the cytoskeleton organization at the cell interface between pDCs and DENV infected cells was determined by confocal microscopy analysis. We observed an accumulation of the actin network at the cell contacts (Figure 7A–E), while the microtubule network was not markedly modified at this location (Figure S11A, left panel). In agreement with the importance of secreted structural components for pDC activation (Figure 6), specific immunostaining of non-permeabilized cells revealed that envelope proteins (i.e., E GP and prM) were both present as clusters at the interface between pDCs and infected cells (Figures 7F–Q and S12).

**Figure 8:**
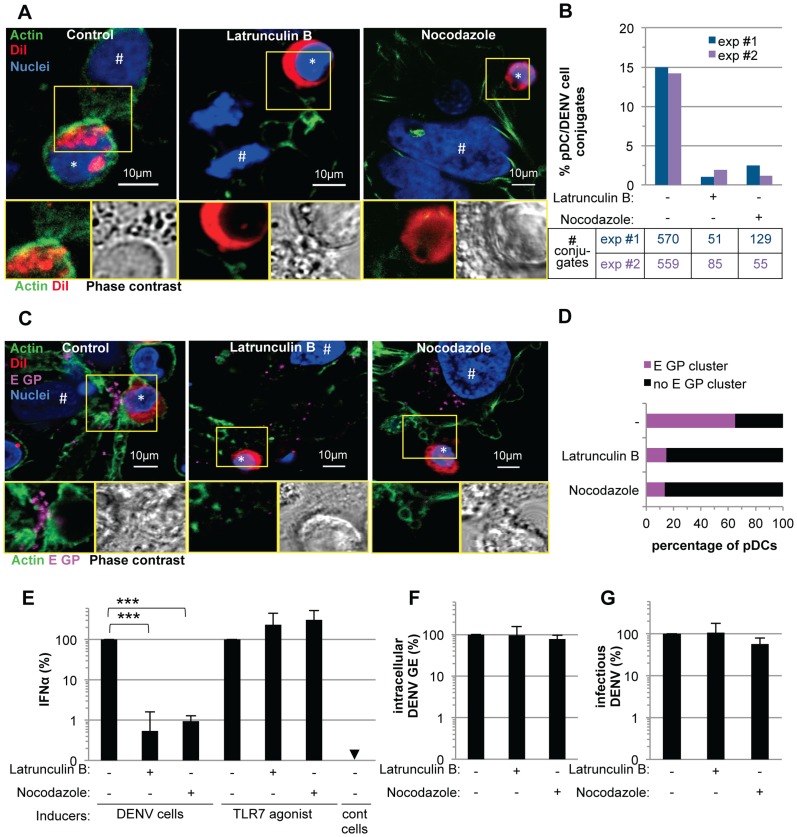
pDC IFNα production triggered by DENV infected cells is modulated by actin cytoskeleton network. (**A**) Imaging of actin network in co-cultures of pDCs and DENV infected cells treated, or not (control), with cytoskeleton inhibitors (Latrunculin B and Nocodazole, 1 µM). Upper panels, confocal microscopy analysis of actin (green), DiI-stained pDC (red, *) and nuclei (blue). # indicates Huh7.5.1 cells. Lower panels, magnification of yellow-boxed cell contacts with actin-DiI (Left) and phase contrast (Right). Representative of 3 independent experiments. (**B**) Quantification of the conjugates between CD123-stained pDCs and GFP-expressing DENV infected Huh7.5.1 cells analyzed by imaging flow cytometry in co-cultures treated with inhibitors as in (A). Results are expressed as percentages of pDC/DENV cell conjugates *i.e.*, conjugates of at least one cell solely CD123+ and one cell GFP+, relative to the total number of single cells (GFP+ or CD123+) and the conjugates, 2 independent experiments (exp #1 and #2). Summary table, number of recorded conjugates in each individual experiment. (**C**) Imaging of E GP clustering in co-cultures treated as in (A). Upper panels, confocal microscopy analysis with actin (green), DiI-stained pDC (red), E GP (purple, immunodetection without permeabilization step) and nuclei (blue). Lower panels, magnification of yellow-boxed cell contacts with actin-E GP (left) and phase contrast (right). (**D**) Quantification of E GP clustering at the pDC/DENV infected cell interface. Results are expressed as percentages of pDCs with E GP clustering at the interface with DENV infected cells relative to the total number of pDCs. pDC included in the analysis were in close proximity to E GP+/DiI- Huh7.5.1 cells. Similar results were obtained in 3 independent experiments, ≈20 pDCs observed per experimental condition by two blind visual examinations. (**E**) Quantification of IFNα in the supernatants of pDCs triggered by co-cultured DENV infected Huh7.5.1 cells (DENV cells) or by TLR7 agonist. Co-cultures were treated with inhibitors as in (A). Results are expressed relative to IFNα produced in the absence of inhibitor, set to 100 (means ± SD, n = 4; paired Student's t test: ***, p<0.0001). Results of intracellular DENV RNA levels (**F**) and infectious virus production (**G**) by DENV infected cells treated as in (A) relative to levels in the absence of inhibitor, set to 100 (means ± SD, n = 4).

These observations prompted us to define the impact of the cytoskeleton network on cell contact-dependent pDC IFNα production. We showed that two inhibitors of the cytoskeleton network, Latrunculin B and Nocodazole (*i.e.*, actin and microtubule depolymerizing drugs, respectively) disrupted the actin network in co-cultures of pDC/DENV infected cell (Figure 8A), consistent with previous reports [42], [43]. As expected, the microtubule network was only perturbed by Nocodazole treatment (Figure S11A) [44]. By imaging flow cytometry analysis of GFP expressing-DENV infected cells co-cultured with pDCs (stained by pDC marker CD123) (Figures S13), we showed that the frequency of conjugates between pDCs and DENV infected cells was greatly decreased by inhibitors of the cytoskeleton network (Figure 8B and S13). Both these inhibitors, in conjunction with the loss of actin accumulation at the contacts (Figure 8A), impaired E glycoprotein clustering (Figure 8C). Indeed, quantifications performed in a “double-blind” set-up revealed that, while E GP clustering was readily observed at the cell interface in untreated co-cultures (*i.e.*, ≈60% of the pDCs at close proximity with DENV infected cells harboring E GP clustering), these frequencies were reduced to 15% for co-cultures treated with either inhibitor (Figure 8D). Importantly, similar treatments inhibited IFNα production by the pDCs (Figure 8E). Neither compound inhibited DENV RNA replication in the infected cells and infectious viral production (Figures 8F–G), nor did they prevent the internalization ability of pDC, as assessed by membrane dye uptake (Figure S11B). In addition, they did not inhibit pDC IFNα production triggered by a TLR7 agonist (Figure 8E), thus ruling out potential nonspecific effects of these compounds on pDC responsiveness.

Altogether, these results suggested that the cytoskeleton-dependent regulation of cell contacts and apposed GP clustering likely favors the subsequent activation of IFNα production by the pDCs.

### Cell producing immature DENV particles trigger pDC IFNα production more potently than cells releasing mature virus

The phenotypic analysis of a virus production defective mutant (*i.e.*, P217A) (Figure 6) revealed that infectious virus production is not required and/or is not rate-limiting for pDC activation.

Like many viruses, DENV infected cells release immature non-infectious particles harboring uncleaved precursor membrane proteins (prM), that are generated by inefficient cleavage of prM by the resident trans-Golgi protease furin [21], [22], [23], [24], [25], [26]. To determine whether immature particles can serve as vehicles from DENV infected cells to contacting pDCs, we first determined the presence of prM protein dots inside co-cultured pDCs by using an antibody recognizing the pr peptide [45] and by examining consecutive Z-sections by confocal microscopy analysis. Dots of prM were observed inside pDCs co-cultured with DENV infected cells (Figures S14A and S14C) with very little background staining in pDCs co-cultured with uninfected control cells (Figures S14B and S14C), suggesting that prM (and/or pr peptide), along with the E GP (Figures 6E, S8 and S9) are readily transferred to the pDCs.

**Figure 9:**
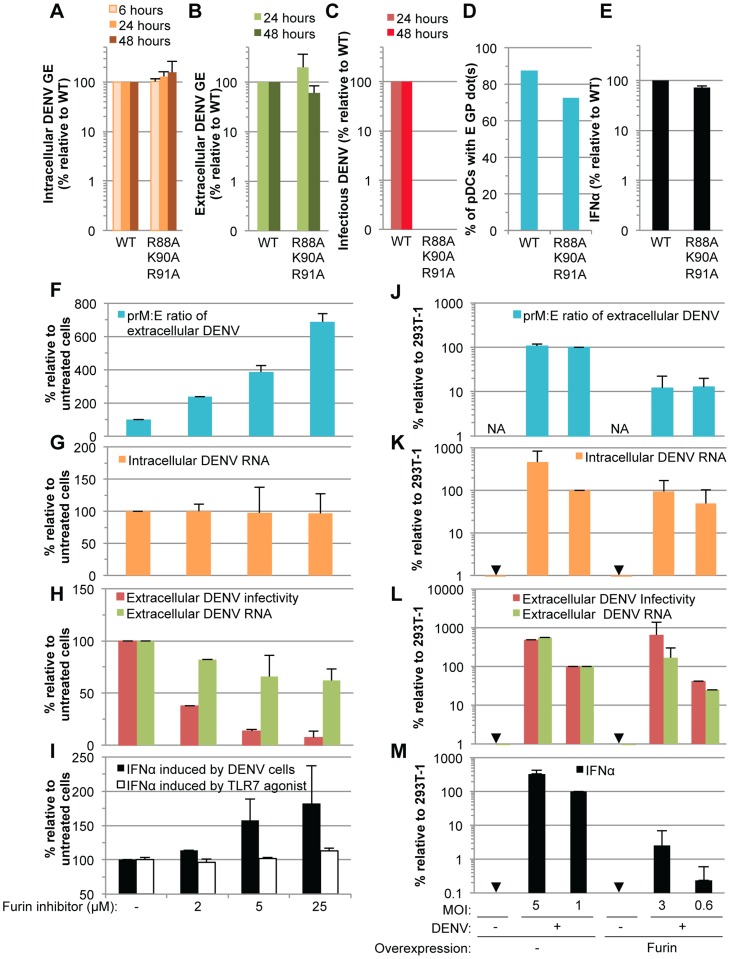
Comparison of pDC IFNα production triggered by mature versus immature DENV particles. (**A–E**) The impact of mutations in prM (substitutions R88A, K90A and R91A) on the levels of intracellular DENV RNA (**A**), extracellular DENV RNA (**B**) infectious virus production **(C**), transmission of E GP from cells harboring the WT and mutant DENV genomes to pDCs (**D**) and quantification of IFNα secretion by pDCs (**E**) analyzed as described in the legend of Figure 6. Results of 3 independent experiments in triplicates are expressed as percentages relative to the WT DENV genome (error bars represent means ± SD). (**F–I**) The impact of a furin inhibitor, at the indicated concentrations, on the prM∶E ratios in extracellular viral supernatants detected by ELISA (**F**), levels of intracellular DENV RNA (**G**), extracellular DENV RNA and infectious virus production (**H**), and quantification of IFNα secretion by pDCs triggered by co-cultured with DENV infected cells or incubation with TLR7 agonist (R848 at 50 ng/mL) (**I**). Results are expressed relative to the untreated cells (-), set at 100 (error bars represent the means ± SD, 3 independent experiments in triplicates). (**J–M**) The impact of furin overexpression, or not (-), in 293T cells infected by DENV at different MOIs, as indicated. The panels are displayed as in (**F–I**), with extracellular prM∶E ratios (**J**), intracellular DENV RNA (**K**), extracellular DENV RNA and infectious virus production (**L**), and quantification of IFNα secretion by co-cultured pDCs (**M**). Results are expressed relative to 293T cells infected at a MOI of 1 in absence of Furin overexpression (indicated as 293T-1), set to 100 (error bars represent the means ± SD, 3 independent experiments in triplicates). Arrows indicate results below the detection limit of assay as described in the legend of Figure 6. NA; Not Applicable.

Next, to determine the ability of immature particles to convey immunostimulatory RNAs to pDCs, we tested the effects of DENV genomes encoding mutations in the furin cleavage site of the prM protein (*i.e.*, the substitutions R88A, K90A and R91A), which, as expected from previous reports with single mutations [26], failed to produce infectious virus (Figure 9C). By contrast, RNA replication, intracellular viral protein expression (Figures 9A, S7A and S7B), release of viral components (Figures 9B and S7C), and transmission of viral components to the pDCs (Figures 9D and S8D) were maintained at levels comparable to the WT counterparts. Remarkably, the pDCs produced similar levels of IFNα when comparing contacting cells producing non-infectious immature virions *vs* WT DENV (Figure 9E). Similar results were obtained when using various concentrations of cells harboring WT/mutant DENV genome (Figure S7D). Therefore, these results suggested that cells producing immature particles potently trigger IFNα production by contacting pDCs.

Next, to define the specific function of uncleaved prM-containing particles in pDC activation by DENV infected cells, we designed experiments aiming at modulating the levels of prM maturation. Firstly, we assessed the impact of an inhibitor of furin. As expected, this inhibitor markedly decreased the maturation of DENV particles, as shown by an increased prM∶E ratio measured by ELISA (Figure 9F). The production of extracellular infectious DENV was also reduced in a dose-dependent manner upon furin treatment (Figure 9H), while the levels of intracellular DENV RNA were unchanged (Figure 9G). Remarkably, inhibition of prM cleavage enhanced IFNα productions by co-cultured pDCs in a dose-dependent manner (Figure 9I). Increased pDC activation was observed despite a reduction in the release of physical particles, as shown by extracellular DENV RNA measurement (Figure 9H). Altogether these results suggested that the activation of pDCs triggered by contacting infected cells inversely correlates with the levels of prM maturation.

To further confirm these results, we studied the impact of furin up-regulation. As expected, cells overexpressing furin produced viral particles containing reduced prM∶E ratios (*i.e.*, ≈10-fold reduction) (Figure 9J). The specific infectivity of DENV particles was increased upon furin overexpression (*i.e.*, ≈3-fold increase in the ratios of infectivity to extracellular DENV RNA, comparing furin-overexpressing cells to counterpart control cells). Thus, cells overexpressing furin were compared to counterpart cells that produced either similar levels of intracellular and/or extracellular DENV RNA, or alternatively, similar production of infectious virus, by using different MOIs (Figure 9K–L). Our results indicated that cells producing more mature particles were clearly impaired at triggering IFNα production by co-cultured pDCs (Figure 9M).

Altogether these results demonstrated that cells producing immature DENV particles are very potent at inducing IFNα production by pDCs, as compared to cells releasing mature virions.

## Discussion

DENV has rapidly emerged in recent years as the most significant arboviral disease of humans, with greater than half of the world population at risk of infection [46]. Despite many years of research, the virus–host interactions that determine dengue pathogenesis are still incompletely understood [47]. Nonetheless, the self-limiting febrile symptoms observed in most DENV-contracted cases and the short course of illness suggest a key role for innate immune defenses in controlling DENV infection at early stages [18]. Accordingly, *in vivo* studies have demonstrated a critical role for type I IFNs in the host defense against DENV [16], [18], [19]. Furthermore, the activation of pDCs strongly correlates with the disease outcome of DENV infected patients [48]. Importantly, a study of children with DENV infections across a broad range of illness severities suggested that a blunted blood pDC response to systemic infection was associated with higher viremia levels and was a key step in the pathogenic cascade toward severe disease [49]. Although the activation mechanism and exact function are still elusive, altogether, these findings highlight the critical roles played by pDCs and the IFN response on disease progression in DENV infected individuals. Here, we revealed that DENV infected cells potently trigger IFNα secretion by non-permissive pDCs, a host response that bypasses the evasion from the innate response within infected cells. Furthermore, we demonstrated that TLR7-dependent IFNα production by pDCs in response to infected cells is concurrent with other hallmarks of innate immunity, such as inflammatory cytokine secretion, ISG up-regulation and pDC maturation.

In agreement with our results, Rodriguez at al. showed that DENV-containing supernatants failed to trigger pDC IFNα production [50], while other reports suggested that they triggered pDC activation [48], [51]. This discordance may be explained by the preparation and concentration of supernatants and large number of pDCs that were used in the latter reports. Remarkably, the results of our study demonstrated that, despite continuous exposure to the infected cell milieu, physical separation from infected cells precludes the IFN response by pDCs. Consistently, strong pDC IFNα secretion was induced by co-cultured DENV infected cells (*i.e.*, up to 0.5 µg/ml), indicating that cell-to-cell contact is a key feature of pDC activation. Interestingly, cell-to-cell transmission of immunostimulatory signals appears to be a common characteristic of pDC induction, as shown in this report for two members of the *Flavivirus* genus, DENV and WNV and as previously reported for other viruses, *i.e.* HCV, HIV, LCMV and CSFV [3], [4], [5], [6], [7]. Specifically, our previous results obtained in the context of HCV indicated that pDC stimulation occurs *via* viral RNA-containing exosomes. In this context, we suggested that the concentration of immunostimulatory exosomes in the supernatants was below an activating threshold for pDC stimulation, while this threshold might be reached in the intercellular space when cells are in contact [3].

Importantly, we showed here that viral structural components are detected in clusters at the interface between pDCs and infected cells. This finding suggests that cellular surface molecules and/or structures might concentrate the PAMP-carrier at the cell contacts, thereby enhancing transmission to pDCs. We further revealed that the actin network is pivotal for both this clustering of viral components at the pDC-infected cell interface, likely by regulating cell-to-cell contacts, and for pDC activation. Based on this observation, it is conceivable that the cytoskeleton structure serves as a platform contributing to the cell-to-cell transmission of viral components to the pDCs. Additional experiments will be required to test these hypotheses and to determine whether, for the various viruses that trigger the pDC IFN response in a cell-to-cell contact dependent manner, the mechanism of activation involves either common or distinct cellular factors and/or structures at the contacts.

The mechanism we have identified is distinct from the conventional induction of the innate response, which typically occurs by the recognition of viral nucleic acids within infected cells [1], [2]. Moreover, in contrast with the previously characterized induction of pDC IFNα production through contact with infected cells [3], [4], [5], [7], here we have defined a sensing pathway, which requires an E glycoprotein-dependent secretion of viral components, notably viral RNA, to trigger the pDCs. As such, it is different from the mechanism of induction by cells infected by other viruses, which does not require viral structural proteins [3], [4], [5], [7]. Indeed, our results illustrate the crucial role of DENV envelope proteins in the induction of the innate response by neighboring IFN producer pDCs that are not permissive to infection. Importantly, our results revealed that cells producing uncleaved prM-containing immature particles triggered IFNα by pDCs more potently than cells efficiently producing fully mature virions. These immature particles are known to be deficient for the membrane fusion step, which occurs in the endo-lysosomal compartment during cell entry [52], [53], [54]. Interestingly, recognition of viral RNA by TLR7 sensor also takes place in this cellular compartment [1], [55]. Therefore, based on these findings, we suggest a working model in which an extended retention within the endosomal compartment of fusogenic-deficient immature particles may favor the exposure of their viral genome for TLR7 recognition. In contrast, mature virions, which are fusion-competent, could escape from this compartment by membrane fusion. Additional experiments will be required to firmly validate and generalize this new concept.

Several reports have demonstrated that a large proportion of uncleaved prM-containing immature particles are released from DENV infected cells, *i*.e., 30-to-40% of viral particles [21], [22], [23], [24], [25], [26], on which prM content is variable on a per-particle basis [56], [57]. Consistently, we showed that furin overexpression reduced the levels of immature virus, otherwise, produced by DENV infected cells, and concomitantly with reduced pDC IFN response. Although direct proof is still required, current evidence supports the *in vivo* existence of uncleaved prM-containing virus. Previous studies have demonstrated that a proportion of B cells isolated from DENV infected individuals produces monoclonal antibodies against prM [58], [59]. In addition, the characterization of these anti-prM antibodies indicated that they are a major component of the serological response to DENV infection, leading to increased replication in Fc receptor-bearing cells *via* antibody-dependent enhancement (ADE) [56], [58], [60]. Importantly, our results illustrate a previously unsuspected function of these immature particles in innate immunity in mediating an IFN response by non-permissive bystander pDC. Indeed, the results of our study imply that the suboptimal furin-cleavage sequence, likely evolutionarily conserved to favor efficient export of infectious virus by preventing premature membrane fusion in the secretory pathway and cell entry of immature virus into Fc-receptor-expressing cells by ADE [21], [22], [23], [27], [28], might also, by producing an IFN-inducer, contribute to regulate dengue pathogenesis. It is possible that pDC activation by infected cells elicits a strong local innate response that may lead to viral replication suppression or, alternatively, to the possible subsequent recruitment of DENV permissive cells and systemic viral spread. It is also conceivable that the interplay between pDCs and other cells regulating the innate responses, in turn, modulates this newly identified innate sensing mechanism of infected cells and/or the homing of pDCs to the infection site.

Productive infection of cells with a wide range of enveloped viruses depends critically on the processing of the viral surface glycoproteins by cellular proteases [20]. Yet, depending on viral variants/strains, such cleavages might be limited by the differential requirement for certain host proteases, as their expression can be tissue-restricted. These selective requirements may contribute to their virulence, as proposed for influenza virus [61]. Additionally, suboptimal cleavage sites are evolutionarily maintained by sequence features, such as, *e.g.*, the presence of acidic residues or glycosylation sites adjacent to the cleavage site [23], [62]. These events lead to the release of viral particles with uncleaved glycoproteins, as shown for viruses such as, *e.g.* measles virus [63], influenza virus [61], [64], DENV and WNV [53], [56]. Therefore our results, by uncovering a functional role of immature viral particles in innate immunity, may have broad implications for our understanding of the host-virus relationship.

## Materials and Methods

### Biological materials

Huh-7.5.1 [65], Vero E6 (ATCC CRL-1586), Hela (ATCC CCL-2) and HEK-293T (ATCC CRL-1573) cells were maintained in Dulbecco's modified Eagle medium (DMEM) (Life Technologies) supplemented with 10% FBS, 100 units (U)/ml penicillin, 100 mg/ml streptomycin, 2 mM L-glutamine and non-essential amino acids (Life Technologies) at 37°C/5% CO_2_. BHK-21 cells (ATCC CCL-10) were maintained in Eagle's MEM (Life Technologies) with the same supplements. pDCs were isolated from 450 ml of blood from healthy adult human volunteers which was obtained according to procedures approved by the “Etablissement Français du sang” (EFS) Committee. PBMCs were isolated using Ficoll-Hypaque density centrifugation. pDCs were positively selected from PBMCs using BDCA-4-magnetic beads (MACS Miltenyi Biotec) and cultured as previously described [3]. Monocytes were positively selected from pDC-depleted PBMCs using CD14-magnetic beads (MACS Miltenyi Biotec) according to the manufacturer's instructions, with a typical purity of 95% of CD11c-positive cells. CD14+ cells were then differentiated to monocyte-derived DCs (mo-DCs) by incubation for 6 days in RPMI 1640 medium supplemented with 10% FBS, 100 U/ml penicillin, 100 mg/ml streptomycin, 2 mM L-glutamine, non-essential amino acids, 1 mM sodium pyruvate and 0.05 mM βmercaptoethanol (Sigma-Aldrich) with 500 U/ml human granulocyte-macrophage colony-stimulated factor (GM-CSF) and 2,000 U/ml human interleukin 4 (IL-4) (MACS Miltenyi Biotec), as previously described [12]. To generate monocyte-derived macrophages, monocytes were cultured in the same medium as for the mo-DCs with 500 U/ml GM-CSF for 6 days.

### Reagents

The antibodies used for immunoblotting were mouse anti-E glycoprotein (4G2 and 3H5) kindly provided by P. Despres (Pasteur Institut, Paris, France); mouse anti-capsid (6F3) kindly provided by J. Aaskov (Queensland University of Technology, Brisbane, Australia); mouse anti-actin (AC74, Sigma Aldrich). The antibodies used for immunostaining were mouse PE-conjugated anti-CD123, mouse APC-conjugated anti-BDCA-2, mouse APC-conjugated anti-IFNα (MACS Miltenyi Biotec), mouse PerCP-conjugated anti-CD83 (eBioscience), and mouse APC-conjugated anti-CD86 (BD Bioscience), mouse anti-DENV prM (clone DM-1, Abcam), mouse anti-alpha tubulin (DM1A, Sigma Aldrich); Ficoll-Hypaque (GE Healthcare Life Sciences); LPS, TLR7 agonist (R848) and TLR9 agonist (ODN2216) (Invivogen); TLR7 antagonist, IRS661 (5′-TGCTTGCAAGCTTGCAAGCA-3′) synthesized on a phosphorothionate backbone (MWG Biotech); Fc Blocking solution (MACS Miltenyi Biotec); Golgi-Plug and permeabilization-wash solution (BD Bioscience); IFNα and IFNβ ELISA kit (PBL Interferon Source); IL-6 and TNFα ELISA kit (Affymetrix, eBioscience); Lipofectamine 2000 (Life Technologies); 96-well format transwell chambers (Corning); LabTek II Chamber Slide System, 96-Well Optical-Bottom Plates and Nunc UpCell 96F Microwell Plate (Thermo Fisher Scientific); CF488A-conjugated phalloidin (Biotium); Vibrant cell-labeling solution (CM-DiI, Life Technologies); Hoescht and Alexas-conjugated secondary antibodies (Life Technologies); iScript cDNA synthesis kit (Biorad), qPCR kit (Life Technologies). Latrunculin B, nocodazole, chlorpromazine, dynasore and Gö6983-PKC were purchased from Sigma-Aldrich.

### Preparation of viral stocks and infection

Viral stocks of the prototypic DENV-2 strain New Guinea C (NGC) (AF038403) were produced using *in vitro* RNA transcripts prepared from DENV-2 infectious plasmid clone pDVWS601 plasmid [66] linearized with XbaI and using mMESSAGE mMACHINE T7 Kit (Ambion). *In vitro* transcribed RNA was introduced into BHK-21 cells by electroporation as previously described [31]. Briefly, 5 µg of *in vitro* transcribed RNA was used to transfect 4×10^6^ cells by electroporation. Six hours post-transfection, the culture medium was refreshed. Virus containing supernatants collected at 3 days post-electroporation were clarified through a 0.45 µm filter (Corning).

Viral stocks of WNV (lineage II, strain 956 D117 3B) [67] were produced by transfection of 2×10^6^ HEK-293T cells with 4 µg of the plasmid pWNII-GFP [67] using the Lipofectamine 2000 transfection reagent (Invitrogen) in optiMEM. Six hours post-transfection, the medium was refreshed with HEK-293T culture medium. Virus containing supernatants collected 48 h post-transfection were clarified through a 0.45 µm filter (Corning).

Viral stocks of vesicular stomatitis virus (VSV-GFP, infectious titer of ≈10^9^ Tissue Culture Infectious Dose (TCID50)/ml) were produced as previously described [68] and kindly provided by Dr J. Perrault (Department of Biology, Center for Microbial Sciences, San Diego State University, CA, US). Viral stocks of Influenza A Virus (FluAV, A/WSN/33 strain, delta NS1, *i.e.*, infectious titer of ≈10^6^ plaque forming unit (PFU)/ml) were produced as previously described [69] and kindly provided by Dr R. Le Goffic (Unite de Virologie et Immunologie Moleculaires, Jouy-en-Josas, France). Sixteen hours prior to co-culture with pDCs, Huh7.5 cells, which are known to be deficient for the RIG-I signaling pathway [70], were used to rule out the confounding contribution of IFNα production by the infected cells themselves and infected at MOI of 0.1 and 0.5 for VSV-GFP and FluAV, respectively.

### Introduction of mutations into the genomic length DENV-2 strain NGC cDNA clone pDVWS601

Introduction of mutations into the genomic length DENV-2 strain NGC cDNA clone pDVWS601, encoding amino acid substitutions into the E glycoprotein (*i.e.*, H244A, D215A, P217A) and NS5 (*i.e.*, Rep^−/−^, containing the multiple amino acid substitutions G81A, G83A and G85A) have been described previously [31], [32]. Mutations encoding amino acid substitutions in prM (amino acids R88A/K90A/R91A) and an in frame four amino acid deletion in the capsid (amino acids V51-to-L54) were first introduced into DENV-2 subgenomic cDNA fragments by overlap-PCR (OL-PCR) using mutagenic primers. The sequences of the primers are described in the Table S1. The OL-PCR fragments were purified and cleaved with *Bsr*GI and *Sph*I and then transferred into the pDVWS601 plasmid that had been cleaved with the corresponding restriction enzymes. The presence of the mutations and sequence of the PCR derived regions were confirmed by sequencing.

*In vitro* RNA transcripts were prepared from the parental and mutated pDVWS601 plasmids as described above and transfected into Huh7.5.1 cells using the Lipofectamine 2000 transfection reagent (Life Technologies), according to the manufacturer's instruction. One µg of RNA was used to transfect a 60% confluent cell monolayer contained in a single well of a 6-well plate following the manufacturer's protocol. Six hours post-transfection, the cells were either harvested for the quantification of viral RNA (6 hour time point) or washed 3 times with PBS and fresh culture medium added to the cells for additional incubation times. At 24 and 48 hours post-transfection, the cells were harvested for the determination of RNA and protein levels and their supernatants collected for the quantification of viral RNA and infectious titer or concentrated by ultracentrifugation for the determination of protein levels by Western blot. At 24 hours post-transfection, the cells were harvested and co-cultured with isolated pDCs for 18–20 hours.

### Co-culture experiments

Forty-eight hours prior to co-culture, cells were infected at a MOI of 3 using a viral stock of WNV or DENV. Unless otherwise indicated, 2×10^4^ pDCs were co-cultured with 10^5^ infected cells, transfected cells or uninfected parental cells, or treated with 100 µl of supernatant from the latter cells in a 200 µl final volume in 96-well round-bottom plates incubated at 37°C/5% CO_2_. Eighteen to twenty hours later, cell-culture supernatants were collected and the levels of IFNα, IFNβ, TNFα and IL-6 were measured using a commercially available ELISA kits specific for IFNα and IFNβ (PBL Interferon Source), TNFα and IL-6 (Affymetrix), following the manufacturer's instructions. When indicated, 10^5^ infected cells or uninfected cells were co-cultured with 3×10^4^ pDCs or with 10^5^ naïve recipient cells, as indicated, in 96-well format transwell chambers separated by a 0.4 µm membrane (Corning).

### Immunostaining and FACS analysis

At the indicated times, cells were harvested and resuspended using 0.48 mM EDTA-PBS solution (Life Technologies). After incubation with Fc receptor blocking reagent (MACS Miltenyi Biotec) for 10 minutes at 4°C, surface staining of pDC markers, CD123 and BDCA-2 and/or the cell differentiation markers CD83 and CD86 were detected by a 40 minute incubation at 4°C with 5 µg/mL of the indicated combinations of PE-conjugated mouse anti-CD123, APC-conjugated anti-BDCA-2, PerCP-conjugated anti-CD83, and APC conjugated anti-CD86, respectively, diluted in staining buffer (PBS without calcium and magnesium, with 2% FBS), followed by PBS washes. Cells were then fixed by incubation for 20 minutes at room temperature with 4% paraformaldehyde, followed by 20 minutes incubation with 0.1 M glycin-PBS at room temperature and two PBS washes. For intracellular-immunostaining of IFNα, cocultivated cells were treated with 1 µl/ml GolgiPlug solution (BD Bioscience) before collection. After fixation and CD123-staining steps, IFNα was detected by a 40 minute incubation with APC-conjugated mouse anti- IFNα (MACS Miltenyi Biotec) diluted at 1∶10 in permeabilization buffer (BD Bioscience). Cells were then washed twice with permeabilization buffer and resuspended in staining buffer. Flow cytometric analysis was performed using a Digital LSR II, and the data were analyzed with Flow Jo software (Tree Star). The corresponding control isotypes served to define the specific signal.

### Detection of DENV RNA in pDC-infected cell co-cultures by Fluorescent *In Situ* Hybridization (FISH) analysis

After isolation, 5×10^4^ pDCs were stained by using 0.5 µM Vibrant cell-labeling solution (CM-DiI, Life Technologies) by successive incubations for 10 and 15 minutes at 37°C and 4°C respectively. Labeled pDCs were washed twice with PBS and then co-cultured with pre-plated DENV infected cells for 5 hours at 37°C in glass bottom 96 well-plate (Fisher Scientific), pretreated with poly-L-lysine at 8 µg/mL. After 4% PFA fixation at room temperature and PBS washing, DENV plus strand RNA was detected using a probe set that targets a region between nucleotide positions 8437-to-9685 in the DENV-2 NGC genome (Panomics/Affymetrix) according to the manufacturer's instructions. For IFNα immunostaining, the cells were permeabilized by incubation for 7 minutes in PBS containing 0.3% (v/v) Triton - and 3% (w/v) BSA, then incubated with mouse anti-IFNα antibody (MACS Miltenyi Biotec) at 2 µg/ml in PBS containing 3% BSA for 40 minutes at room temperature, followed by an incubation with Alexa 647-conjugated anti-mouse antibody (Life Technologies) and Hoechst dye for 40 minutes at room temperature. As controls, FISH detection of DENV RNA were performed in co-cultures of pDCs with non-infected cells and in co-cultures of pDCs with DENV infected cells by omitting DENV-specific probe and by following the same procedure of hybridization and immunostaining. Images were acquired with a Zeiss LSM 710 laser scanning confocal microscope and analyzed with Image J (http://rsb.info.nih.gov/ij) and IMARIS (Bitplane Inc.) software packages.

### Immuno-detection of DENV E and prM surface protein and confocal analysis

After immune-isolation, pDCs were stained with 0.5 µM Vibrant cell-labeling solution (CM-DiI) as above-described. 4-to-5×10^4^ DiI-labeled pDCs (DiI-pDCs) were co-cultured with 4-to-5×10^4^ DENV infected Huh7.5.1 cells for 8 hours at 37°C. For analysis of DENV E and prM transfer into pDCs and cell contacts, co-cultures were performed in LabTek II Chamber Slide System (Nunc). After 4% PFA fixation and three PBS washes, cells were permeabilized 7 min with 0.1% Triton in PBS prior immunostaining. For analysis of DENV surface protein clustering at the cell contacts, co-cultures were incubated in a 96-Well Optical-Bottom Plates. After 4% PFA fixation and three PBS washes, immunostainings were performed without permeabilization step, as previously described [71]. After blocking step (PBS 3% BSA) actin filaments were stained with CF488A-conjugated phalloidin (Biotium) at 1.25 U/mL, α-tubulin was stained with mouse anti-α tubulin (DM1A clone, from Sigma) at 1∶2000-dilution, DENV E glycoproteins were detected using anti-E antibody (3H5 clone) at 1∶500-dilution and anti-PrM antibody (DM-1 clone, Abcam) at 1∶50-dilution and IFNα was detected by a mouse anti-IFNα (Miltenyi) at 1∶20-dilution. Antibodies were diluted in 3% BSA-PBS and added to the cell for 1 hour incubation at room temperature. After three PBS-washes with PBS, cells were incubated with an Alexa 647-conjugated-anti-mouse antibody (for detection of anti-α-tubulin and anti-E antibodies) or Alexa 488-conjugated-anti-mouse (for detection of anti-PrM antibody) at 1∶1000-dilution in 3% BSA-PBS, added to the cells along with Hoechst diluted at 1∶500 (Molecular Probes) for 1 hour incubation at room temperature. After three washes with PBS, cells in 96 wells plate were directly observed and cells in Labtek were mounted with mowiol prior observation. Images were acquired with a Zeiss LSM 710 laser scanning confocal microscope and analyzed with Image J (http://rsb.info.nih.gov/ij) and IMARIS (Bitplane Inc.) software packages.

### Analysis of intracellular and extracellular RNA levels

RNAs were isolated from cells or supernatants harvested in guanidinium thiocyanate citrate buffer (GTC) by phenol/chloroform extraction procedure as previously [3]. The efficiency of RNA extraction and reverse transcription-real-time quantitative PCR (RT-qPCR) was controlled by the addition of carrier RNAs encoding Xef1α (xenopus transcription factor 1α) *in vitro* transcripts in supernatants diluted in GTC buffer. DENV RNA and Xef1α and glyceraldehyde-3-phosphate dehydrogenase (GAPDH) mRNA levels were determined by RT-qPCR using an iScript RT kit (Biorad) and a One-Step PCR Master Mix kit for qPCR and analyzed using StepOnePlus Real-Time PCR system (Life Technologies). The sequences of the primers used for the RT-qPCR are described in Table S1. Extracellular and intracellular DENV RNA levels were normalized for Xef1α and GAPDH RNA levels, respectively.

### Analysis of extracellular infectivity

Infectivity titers in supernatants were determined by end-point dilution using Huh 7.5.1 cells. Foci forming unit (ffu) were detected 72 hours after infection by GFP expression for WNV and anti-E glycoprotein specific immunofluorescence for DENV. Briefly, Huh 7.5.1 cells were fixed with 4% PFA and permeabilization by incubation for 7 minutes in PBS containing 0.1% Triton. Cells were then blocked in PBS containing 3% BSA for 15 minutes and incubated for 1 hour with mouse anti-E glycoprotein (clone 3H5) hybridoma supernatant diluted at 1∶200 in PBS containing 1% BSA. After 3 washes with PBS, cells were incubated 1 hour with secondary Alexa 555-conjugated anti-mouse antibody (1∶1'000-dilution) and Hoechst dye (1∶1'000-dilution) in PBS containing 1% BSA. Percentage of E-positive cells and GFP expressing cells was determined using a Zeiss Axiovert 135 microscope.

### Analysis of concentrated viral supernatants and cell lysats by western blot

Viral supernatant were filtrated through a 0.45 µm filter (Corning) and concentrated prior to Western blot analysis by ultracentrifugation at 110,000× g for 2 hours at 4°C using a SW41 rotor. The pellets were re-suspended in PBS. Viral pellets and cell lysates were extracted using lysis buffer (150 mM NaCl 50 mM Tris HCl pH 8, 1% NP40, 0.5% Deoxycholate, 0.1% Sodium dodecyl sulfate) and analyzed by Western blotting using hybridoma supernatant-containing anti-E (4G2) and anti-capsid (6F3) at the dilution of 1∶500 and actin at 1 µg/ml followed by secondary horse radish peroxidase-coupled antibodies and chemiluminescence.

### Imaging combined with flow cytometry analysis of pDC/DENV cell conjugates

Huh 7.5.1 cells were transduced with retroviral based vector pseudotyped with VSV glycoprotein to stably express GFP, as previously described [72]. Forty-eight hours prior co-culture with pDCs, GFP-expressing Huh 7.5.1 cells were infected at a MOI of 3 using a viral stock of DENV. 10^5^ GFP-expressing DENV infected cells were co-cultured with 3×10^4^ pDCs in low-adherence micro-plate designed for cell harvesting by temperature reduction (Nunc UpCell 96F Microwell Plate from Thermo Scientific) for 5 hours at 37°C in presence, or not, of Latrunculin B and Nocodazole (1 µM), as indicated. After 4% PFA fixation, co-cultured cells were harvested by equivalent multi-pipetting at room temperature and washed three times with staining buffer (PBS without calcium and magnesium with 2% FBS). After incubation with Fc receptor blocking reagent (MACS Miltenyi Biotec) for 10 minutes at 4°C, surface staining of a pDC marker, CD123, was detected by a 40 minute incubation at 4°C with 5 µg/mL of APC-conjugated mouse anti-CD123, diluted in staining buffer, followed by washes with staining buffer. Co-cultured cells were analyzed by Image Stream X technology (Amnis) at magnification ×60 using IDEAS software. The cell population defined as pDC/DENV cell conjugates comprises conjugates of at least one CD123+ cell and at least one cell solely GFP+ cell among the total of APC+ cells, GFP+ cells and conjugates. The cell populations were sorted by using masks (IDEAS software) to eliminate *i/*the non-specific signals *i.e.*, double positive single cells and *ii/*cells with background levels for APC signal. Post-cell sorting, the accuracy of the gated cell population in regards to the defined criteria was controlled by a visual inspection of the individual pictures in the gated cells population (*i.e.*, assessment with 90 randomly picked pictures of the population defined as conjugates). The percentages of gated single cells or conjugates with an accurate phenotype according to the defined criteria among the total of examined pictures per category of cell population were: 97% for GFP+ gated population, 99% for APC-CD123+ gated population and 89% for conjugates.

### Modulation of prM maturation and detection by ELISA

293T cells, which stably express furin, were generated by transfection using Polyethylenimine and selected using hygromycin (at 5 µg/ml). The decRRVKR-CMK inhibitor (Calbiochem) was used to inhibit the Furin activity in Huh7.5.1 co-cultured with the pDCs, as the indicated concentrations. The levels of prM maturation were analyzed by detection of E and prM by ELISA, as previously described [58]. Briefly serial dilutions of viral supernatants were incubated on anti-E (4G2) antibody coated 96-well plate. Then, E and prM were detected by using a humanized version of 3H5 mAb (hu3H5) and anti-prM, respectively. The prM∶E ratios were calculated using the viral supernatant dilution with E detection in the linear range.

### Expression of DENV glycoproteins

DENV-2 NGC prM and E genes were cloned under the control of CMV promoter, by amplification from pSVprME [73] using primers ADVprME_Fwd (GATCCCCGGGACCGCCACCATGGTGAA) and ADVprME_REV (GATCCCCGGGAGCTTGATATCAGGCCTGC) and cloned into the Sma I site of the adenovirus shuttle vector pDC104 under the control of the CMV promoter to produce pAdvprME. AdvprME was transfected into cells using the Xtreme-GENE HP DNA Transfection Reagent, follow the manufacturer's instructions. Six hours post-transfection, the cells were washed with PBS and fresh culture medium added to the cells for additional incubation times. At 48 hours post-transfection, the cells were harvested and co-cultured with isolated pDCs for 18–20 hours. Parallel determination of intracellular protein levels by Western blot in harvested cells and their supernatants concentrated by using vivaspin concentrator with centrifugation at 3000 g for 30 min (cut-off 100 KDa, Sartorius).

### Statistical analysis

Paired Student's t-test was used to analyze data. Data considered significant demonstrated p-values less than 0.05. Data were also analyzed using a two ways non-parametrical analysis of variance (ANOVA), followed by comparison with Levene Test, analyzed with xlstat software. Triangles indicate the experimental conditions that belong to a separated group statistically different from the others.

## Supporting Information

Figure S1 **pDCs robustly produce IFNα in response to DENV infected cells, related to** **Figure 1** **.** pDC depletion or enrichment was performed using an anti-BDCA-4 antibody. (**A**) Representative FACS analysis of pDC depletion and isolation from PBMCs using the pDC selective markers, CD123 and BDCA-2. (**B**) Quantification of IFNα in the supernatants of 10^5^ PBMCs, 10^5^ pDC depleted-PBMCs and 10^3^ isolated pDCs (Responders) co-cultured with DENV infected BHK-21 cells (DENV cells) or treated with 100 µl of supernatants from the latter cells (DENV SN), in a 200 µl final volume. Viral titers in the DENV SN were ≈0.75×10^6^ foci forming units (ffu)/ml. Uninfected BHK-21 cells are referred to as control (cont) cells. Arrows indicate results below the detection threshold of the IFNα ELISA (*i.e.*, 12.5 pg/ml). Results are representative of 3 independent experiments in triplicate. Error bars represent the means ± SD. (**C**) Parallel quantification of IL6 in the supernatants of PBMCs or pDC-depleted PBMCs (Responders) triggered by incubation with LPS (10 µg/mL for 20 hours). Results are representative of 3 independent experiments in triplicates. Error bars represent the means ± SD.
(TIF)

Figure S2 **Effect of infection time duration and of varying the number of infected cells on pDC IFNα secretion, related to** **Figure 1** **.** (**A**) Quantification of IFNα in the supernatants of pDCs co-cultured with DENV cells or incubated with 100 µl of their supernatants (DENV SN) in a 200 µl final volume. Cells were infected at a MOI of 0.2 either 48 or 96 hours prior to co-culture, as indicated. Viral titers of the DENV SN were ≈10^5^ and 4×10^5^ ffu/ml at 48 and 96 hours, respectively. Results of 2 independent experiments in duplicates. Diamonds and histograms represent individual repeats and means, respectively. (**B–C**) Quantification of IFNα in supernatants of pDCs co-cultured with varying numbers of DENV infected cells (DENV cells) (**B**) or WNV infected cells (WNV cells) (**C**), Results of 2 (**B**) and 3 (**C**) independent experiments in duplicates. Diamonds and histograms represent individual repeats and means, respectively. Uninfected cells are referred to as control (cont) cells. Arrows indicate results below the limit of detection of the IFNα ELISA (*i.e.*, 12.5 pg/ml). # cells; number of infected cells.
(TIF)

Figure S3 **Cell-free virus containing supernatants fail to trigger IFNα by pDCs.** Quantification of IFNα in supernatants of pDCs co-cultured with cells infected by DENV cells (**A**) or by WNV (**C**) or inoculated with 100 µl of their supernatants (SN) that were filtrated using 0.45 µm filters (filtration) or not (crude). Arrows indicate results below the limit of detection of the IFNα ELISA (*i.e.*, 12.5 pg/ml). (**B–D**) Parallel determination of infectious viral titers of DENV SN (**B**) and WNV SN (**D**). Results are representative of 3 independent experiments in triplicates. Error bars represent the means ± SD.
(TIF)

Figure S4 **pDCs are not permissive to DENV and WNV infections.** (**A**) Time course analysis of intracellular DENV RNA levels by RT-qPCR. Results are expressed as fold increase relative to the detection at 4 hours post-inoculation with a MOI of 1 for Huh7.5.1 and MOI of 3 for pDCs. Results are expressed as the fold increase of DENV genome equivalents (GE) relative to the 4 hour time point. Results of 2 independent experiments in duplicates. Diamonds represent individual repeats. (**B**) Time course analysis of the percentage of WNV-GFP positive cells after inoculation with WNV-GFP at a MOI of 4 for 293T cells and MOI of 12 for pDCs. Results are quantified by FACS. Results of 2 independent experiments in duplicates. Diamonds represent individual repeats.
(TIF)

Figure S5 **Specificity of the detection of DENV RNA transferred from infected cells to pDCs by RNA FISH assays, related to** **Figure 5** **.** Confocal microscopy analysis of DENV RNA detected by FISH in co-cultures of pDCs with uninfected Huh-7.5.1 cells (Cont cells/pDCs) (**A–B**) and DENV infected Huh-7.5.1 cells with omission of DENV-specific probe (DENV cells/pDCs – no probe) (**C–D**). Cells were co-cultured for 5 hours, followed by the same procedure of hybridization and immunostaining as described in Figure 5A. (**A–C**) Projections of confocal microscopy analysis with DENV RNA (green), DiI-stained pDC (red), IFNα protein (white) and nuclei (blue). Star mark (*) indicates the pDC and hash mark (#) indicates the Huh7.5.1 cell. (**B–D**) Consecutive Z-axis sections with magnification of yellow-boxed pDC, shown in the corresponding panels (A) and (C). Left panels, combined detections of DENV RNA (green), DiI-stained pDC (red), IFNα protein (white). Their individual detections are displayed on the right panels as indicated. Similar results were obtained in 3 independent experiments.
(TIF)

Figure S6 **Induction of ISG expressions by pDCs or PBMCs co-cultured with DENV infected cells.** Quantification of intracellular MxA (**A**) and ISG56 (**B**) mRNA levels of pDCs or PBMCs co-cultured or not with DENV infected Huh7.5.1 cells (DENV cells). Results are expressed as fold increase relative to corresponding co-culture with uninfected Huh7.5.1 (cont) cells. (**C**) Parallel quantification of IFNα in supernatants of pDCs or PBMCs co-cultured or not with DENV infected Huh7.5.1 cells (DENV cells). Results of 2 independent experiments in duplicates. Diamonds and histograms represent individual repeats and means, respectively. Arrows indicate results below the limit of detection of the IFNα ELISA (*i.e.*, 12.5 pg/ml).
(TIF)

Figure S7 **Impact of mutations in DENV NS5, capsid and E and prM on intracellular and extracellular levels of viral proteins, related to** **Figures 6** **and** **9** **.** (**A**) The impact of mutations on levels of intracellular DENV RNAs at the indicated time post-transfection. Results are quantified by RT-qPCR and expressed as DENV genome equivalent (GE)/µg total RNA. (**B–C**) Representative Western blot analyses of intracellular (**B**) and extracellular (**C**) DENV E and capsid protein levels. Detection of the actin protein was used as a loading control. MW; molecular weight markers in kDa. Results are representative of 3 independent experiments. (**D**) Effect of varying numbers of donor cells on pDC IFNα secretion. Quantification of IFNα in supernatants of pDCs co-cultured with varying numbers of cells harboring the WT and mutant DENV genomes (means ± SD, n = 3). Cells harvested at 24 hours post-transfection were co-cultured with pDCs for 20 hours. Uninfected cells are referred to as control (-) cells. Arrows indicate results below the limit of detection of the IFNα ELISA (*i.e.*, 12.5 pg/ml). # cells; number of infected cells.
(TIF)

Figure S8 **Transfer of DENV E glycoproteins from cells harboring the WT and mutant DENV genomes to co-cultured pDCs, related to** **Figures 6** **and** **9** **.** Left panels, representative projections of confocal microscopy analysis of DENV E glycoproteins (E GP, green) detected by immunostaining in co-cultures of pDCs stained by DiI dye (red) and cells harboring the WT genome (**A**), or genome with mutations in E, *i.e.*, substitutions H244A (**B**) and P217A (**C**), with mutations in prM, *i.e.*, substitutions R88A, K90A and R91A (**D**) or cells devoid of DENV genome, as control (**E**); nuclei (blue). Right panels, consecutive Z-axis sections with magnification of yellow-boxed pDC, shown in the corresponding left panels. Cell contours on E GP panels are labeled with dotted lines surrounding DiI staining. Yellow-arrows; E GP dots inside pDC. Star mark (*) and hash mark (#) indicates the pDC and the Huh7.5.1 cell, respectively. Similar results were obtained in 3 independent experiments and summary table of the statistical analysis is displayed in Figure 6E.
(TIF)

Figure S9 **Individual expression of DENV surface proteins (** ***i.e.*** **, E GP and prM) and their transmission into the pDCs are not sufficient to trigger IFNα production.** Representative Western blot analyses of intracellular DENV E and capsid protein levels (**A**) and extracellular DENV E protein levels (**B**) with cells expressing only DENV glycoproteins (GP cells) as compared to DENV infected cells (DENV cells) and uninfected parental cells (Cont cells). Detection of the actin protein used as a loading control, MW; molecular weight markers in kDa. Results are representative of 3 independent experiments. (**C**) Quantification of IFNα in supernatants of pDCs co-cultured with cells expressing only DENV glycoproteins (GP cells) as compared to DENV infected cells (DENV cells) and uninfected parental cells (Cont cells). Arrows indicate results below the limit of detection of the IFNα ELISA (*i.e.* 12.5 pg/ml). Error bars represent the means ± SD, results are representative of 3 independent experiments. (**D**) Representative projections of confocal microscopy analysis of DENV E glycoproteins (E GP, green) detected by immunostaining in co-cultures of pDCs stained by DiI membrane dye (red) with cells expressing DENV glycoproteins (GP cells) as compared to DENV infected cells (DENV cells); nuclei (blue). Star mark (*): pDCs and hash mark (#): Huh7.5.1 cells. (**E**) Consecutive Z-axis sections with magnification of yellow-boxed pDC, shown in the corresponding upper panels. Cell contours on the E GP panels are labeled with dotted lines surrounding DiI staining. Yellow arrows; E GP dots inside pDC. Similar results were obtained in 3 independent experiments. (**F**) Results expressed as the percentages of DiI stained pDCs containing E GP dot(s). Similar results were obtained in 3 independent experiments and ≈20 pDCs, surrounded by at least one E GP positive cell, were observed per experimental condition.
(TIF)

Figure S10 **Impact of internalization inhibitors on IFNα production by pDCs co-cultured with DENV infected cells.** Impact of inhibitors of clathrin-mediated endocytosis (chlorpromazine, CPZ, at 14 µM), of dynamin-dependent internalization (dynasore, at 100 µM) and macropinocytosis (Gö6983-PKC inhibitor, GO, at 5 µM) on pDC IFNα production triggered by DENV infected cells. (**A**) Quantification of IFNα in the supernatants of pDCs co-cultured with DENV infected Huh7.5.1 cells (DENV cells) or, as control, stimulated by TLR7 agonist the R848 (50 ng/mL), an imidazoquinoline known as a cell-permeable weak base that passively diffuses inside the pDCs. Results are expressed relative to IFNα produced in absence of inhibitor, set to 100 (means ± SD, n = 4). Arrows indicate results below the limit of detection of the IFNα ELISA (*i.e.*, 12.5 pg/ml). Quantification of the levels of infectious virus production (**B**) and intracellular DENV genome equivalent (GE) (**C**) by Huh7.5.1 cells infected by DENV at MOI 3 for 48 hours (as for the co-culture in (**A**)) and then incubated, or not, with inhibitors, exactly as in (**A**) (*i.e.* incubation time and concentration). Results are expressed as percentages relative to untreated DENV cells (means ± SD, n = 4).
(TIF)

Figure S11 **Impact of the cytoskeleton inhibitors on microtubule network and FM4-64 internalization, related to** **Figure 8** **.** (**A**) Imaging of immunostained α-tubulin in co-cultures of DiI-stained pDCs and DENV infected cells treated with cytoskeleton inhibitors, exactly as in Figure 8A. Star mark (*): pDCs and hash mark (#): Huh7.5.1 cells. Upper panels, confocal microscopy analysis of α-tubulin (green); DiI-stained pDC (red); nuclei (blue). Lower panels, magnification of yellow-boxed cell contact shown in the corresponding upper pictures with tubulin-DiI staining and phase contrast (left and right panels, respectively). Similar results were obtained in 2 independent experiments. (**B**) Imaging of the internalization of a lipophilic-dye, FM 4-64 (added for 15 min incubation at 37°C) in co-cultures of pDCs and DENV infected cells treated with cytoskeleton inhibitors, exactly as in Figure 8A. Upper panels, confocal microscopy analysis of actin (green); FM 4-64 (red); nuclei (blue). Lower panels, magnification of yellow boxes shown in the corresponding upper pictures, with actin-FM 4-64 and FM 4-64-phase contrast (left and right panels, respectively).
(TIF)

Figure S12 **Specificity of the immuno-detection of DENV E and PrM clustering, related to** **Figure 7** **.** Absence of detection of E glycoprotein (E GP, purple) (**A**) and prM (green) (**B**) in co-cultures of DiI-stained pDCs (red) with uninfected Huh 7.5.1 cells, analyzed exactly as in Figure 7F–K and 7L–Q, respectively. Left panels, confocal analysis of DENV envelope proteins, E GP (purple), prM (green), DiI-stained pDCs (Red), actin detected by Alexa 488-conjugated phalloidin (green), when indicated, and nuclei (blue). Middle panels, confocal microscopy analysis of DENV envelope proteins and nuclei (blue) projected on the phase contrast imaging. Right panels, confocal microscopy analysis of DENV envelope proteins and nuclei (blue). Star mark (*): pDCs and hash mark (#): Huh7.5.1 cells. Similar results were obtained in 3 independent experiments.
(TIF)

Figure S13 **Analysis of the conjugates between pDCs and DENV infected cells by imaging flow cytometry analysis, related to** **Figure 8B** **.** Imaging flow cytometry analysis (ImageStream) of DENV infected Huh7.5.1 cells, which stably express GFP, and co-cultured with pDCs for 8 hours, as described in the Figure 8B. pDCs are detected by the immunostaining of CD123, a pDC specific marker (APC-conjugated anti-CD123 antibody). Representative pictures of the cell population gated as conjugates between pDCs and GFP expressing DENV infected cells (**A**), of the cell population gated as pDCs, single cells (CD123 positive cells) (**B**), and of the cell population gated as DENV infected cells,(GFP positive cells) (**C**). Panels, as displayed from the left to the right, Bright field; GFP field; APC field; GFP/APC field and Merge.
(TIF)

Figure S14 **Transfer of DENV prM from DENV infected cells into co-cultured pDCs.** Left panels, representative projections of confocal microscopy analysis of DENV prM (green) detected by immunostaining in co-cultures of DiI-stained pDCs (red) and DENV infected Huh7.5.1 cells (**A**), or uninfected (Cont) cells (**B**); nuclei (blue). Right panels, consecutive Z-axis sections with magnification of yellow-boxed pDC, shown in the corresponding projection. Cell contours on the DENV prM panels are labeled with dotted lines surrounding DiI staining. Yellow arrows; DENV prM protein dot inside pDC. (**C**) Results expressed as the percentages of DiI stained-pDCs containing prM protein dot(s). Similar results were obtained in 3 independent experiments, with ≈20 pDCs, surrounded by prM positive/DiI negative cell(s) observed per experimental condition.
(TIF)

Table S1 **Sequences of the primers for RT-qPCR and cloning.**
(DOCX)

## References

[1] Kawai T, Akira S (2011) Toll-like receptors and their crosstalk with other innate receptors in infection and immunity. Immunity 34: 637–650. 21616434 10.1016/j.immuni.2011.05.006

[2] Loo YM, Gale M Jr (2011) Immune signaling by RIG-I-like receptors. Immunity 34: 680–692. 21616437 10.1016/j.immuni.2011.05.003 PMC3177755

[3] Dreux M, Garaigorta U, Boyd B, Decembre E, Chung J, et al (2012) Short-range exosomal transfer of viral RNA from infected cells to plasmacytoid dendritic cells triggers innate immunity. Cell Host Microbe 12: 558–570. 23084922 10.1016/j.chom.2012.08.010 PMC3479672

[4] Wieland SF, Takahashi K, Boyd B, Whitten-Bauer C, Ngo N, et al (2014) Human plasmacytoid dendritic cells sense lymphocytic choriomeningitis virus-infected cells in vitro. J Virol 88: 752–757. 24155390 10.1128/JVI.01714-13 PMC3911694

[5] Takahashi K, Asabe S, Wieland S, Garaigorta U, Gastaminza P, et al (2010) Plasmacytoid dendritic cells sense hepatitis C virus-infected cells, produce interferon, and inhibit infection. Proc Natl Acad Sci U S A 107: 7431–7436. 20231459 10.1073/pnas.1002301107 PMC2867703

[6] Lepelley A, Louis S, Sourisseau M, Law HK, Pothlichet J, et al (2011) Innate sensing of HIV-infected cells. PLoS Pathog 7: e1001284. 21379343 10.1371/journal.ppat.1001284 PMC3040675

[7] Python S, Gerber M, Suter R, Ruggli N, Summerfield A (2013) Efficient sensing of infected cells in absence of virus particles by plasmacytoid dendritic cells is blocked by the viral ribonuclease E(rns.). PLoS Pathog 9: e1003412. 23785283 10.1371/journal.ppat.1003412 PMC3681750

[8] Cisse B, Caton ML, Lehner M, Maeda T, Scheu S, et al (2008) Transcription factor E2-2 is an essential and specific regulator of plasmacytoid dendritic cell development. Cell 135: 37–48. 18854153 10.1016/j.cell.2008.09.016 PMC2631034

[9] Reizis B, Bunin A, Ghosh HS, Lewis KL, Sisirak V (2011) Plasmacytoid dendritic cells: recent progress and open questions. Annu Rev Immunol 29: 163–183. 21219184 10.1146/annurev-immunol-031210-101345 PMC4160806

[10] Versteeg GA, Garcia-Sastre A (2010) Viral tricks to grid-lock the type I interferon system. Curr Opin Microbiol 13: 508–516. 20538505 10.1016/j.mib.2010.05.009 PMC2920345

[11] Morrison J, Aguirre S, Fernandez-Sesma A (2012) Innate immunity evasion by Dengue virus. Viruses 4: 397–413. 22590678 10.3390/v4030397 PMC3347034

[12] Aguirre S, Maestre AM, Pagni S, Patel JR, Savage T, et al (2012) DENV inhibits type I IFN production in infected cells by cleaving human STING. PLoS Pathog 8: e1002934. 23055924 10.1371/journal.ppat.1002934 PMC3464218

[13] Yu CY, Chang TH, Liang JJ, Chiang RL, Lee YL, et al (2012) Dengue virus targets the adaptor protein MITA to subvert host innate immunity. PLoS Pathog 8: e1002780. 22761576 10.1371/journal.ppat.1002780 PMC3386177

[14] Anglero-Rodriguez YI, Pantoja P, Sariol CA (2014) Dengue virus subverts the interferon induction pathway via NS2B/3 protease-IkappaB kinase epsilon interaction. Clin Vaccine Immunol 21: 29–38. 24173023 10.1128/CVI.00500-13 PMC3910921

[15] Rodriguez-Madoz JR, Belicha-Villanueva A, Bernal-Rubio D, Ashour J, Ayllon J, et al (2010) Inhibition of the type I interferon response in human dendritic cells by dengue virus infection requires a catalytically active NS2B3 complex. J Virol 84: 9760–9774. 20660196 10.1128/JVI.01051-10 PMC2937777

[16] Simmons CP, Popper S, Dolocek C, Chau TN, Griffiths M, et al (2007) Patterns of host genome-wide gene transcript abundance in the peripheral blood of patients with acute dengue hemorrhagic fever. J Infect Dis 195: 1097–1107. 17357045 10.1086/512162 PMC4042601

[17] de Kruif MD, Setiati TE, Mairuhu AT, Koraka P, Aberson HA, et al (2008) Differential gene expression changes in children with severe dengue virus infections. PLoS Negl Trop Dis 2: e215. 18398488 10.1371/journal.pntd.0000215 PMC2274954

[18] Martina BE, Koraka P, Osterhaus AD (2009) Dengue virus pathogenesis: an integrated view. Clin Microbiol Rev 22: 564–581. 19822889 10.1128/CMR.00035-09 PMC2772360

[19] Sariol CA, Martinez MI, Rivera F, Rodriguez IV, Pantoja P, et al (2011) Decreased dengue replication and an increased anti-viral humoral response with the use of combined Toll-like receptor 3 and 7/8 agonists in macaques. PLoS ONE 6: e19323. 21559444 10.1371/journal.pone.0019323 PMC3084804

[20] Pasquato A, Ramos da Palma J, Galan C, Seidah NG, Kunz S (2013) Viral envelope glycoprotein processing by proprotein convertases. Antiviral Res 99: 49–60. 23611717 10.1016/j.antiviral.2013.04.013

[21] Rodenhuis-Zybert IA, van der Schaar HM, da Silva Voorham JM, van der Ende-Metselaar H, Lei HY, et al (2010) Immature dengue virus: a veiled pathogen? PLoS Pathog 6: e1000718. 20062797 10.1371/journal.ppat.1000718 PMC2798752

[22] Rodenhuis-Zybert IA, Wilschut J, Smit JM (2011) Partial maturation: an immune-evasion strategy of dengue virus? Trends Microbiol 19: 248–254. 21388812 10.1016/j.tim.2011.02.002

[23] Keelapang P, Sriburi R, Supasa S, Panyadee N, Songjaeng A, et al (2004) Alterations of pr-M cleavage and virus export in pr-M junction chimeric dengue viruses. J Virol 78: 2367–2381. 14963133 10.1128/JVI.78.5.2367-2381.2004 PMC369205

[24] Wang S, He R, Anderson R (1999) PrM- and cell-binding domains of the dengue virus E protein. J Virol 73: 2547–2551. 9971841 10.1128/jvi.73.3.2547-2551.1999 PMC104503

[25] van der Schaar HM, Rust MJ, Waarts BL, van der Ende-Metselaar H, Kuhn RJ, et al (2007) Characterization of the early events in dengue virus cell entry by biochemical assays and single-virus tracking. J Virol 81: 12019–12028. 17728239 10.1128/JVI.00300-07 PMC2168764

[26] Junjhon J, Lausumpao M, Supasa S, Noisakran S, Songjaeng A, et al (2008) Differential modulation of prM cleavage, extracellular particle distribution, and virus infectivity by conserved residues at nonfurin consensus positions of the dengue virus pr-M junction. J Virol 82: 10776–10791. 18715923 10.1128/JVI.01180-08 PMC2573171

[27] Pierson TC, Diamond MS (2012) Degrees of maturity: the complex structure and biology of flaviviruses. Curr Opin Virol 2: 168–175. 22445964 10.1016/j.coviro.2012.02.011 PMC3715965

[28] Zellweger RM, Prestwood TR, Shresta S (2010) Enhanced infection of liver sinusoidal endothelial cells in a mouse model of antibody-induced severe dengue disease. Cell Host Microbe 7: 128–139. 20153282 10.1016/j.chom.2010.01.004 PMC2824513

[29] Diebold SS, Kaisho T, Hemmi H, Akira S, Reis e Sousa C (2004) Innate antiviral responses by means of TLR7-mediated recognition of single-stranded RNA. Science 303: 1529–1531. 14976261 10.1126/science.1093616

[30] Lee HK, Lund JM, Ramanathan B, Mizushima N, Iwasaki A (2007) Autophagy-dependent viral recognition by plasmacytoid dendritic cells. Science 315: 1398–1401. 17272685 10.1126/science.1136880

[31] Kroschewski H, Lim SP, Butcher RE, Yap TL, Lescar J, et al (2008) Mutagenesis of the dengue virus type 2 NS5 methyltransferase domain. J Biol Chem 283: 19410–19421. 18469001 10.1074/jbc.M800613200

[32] Kroschewski H, Sagripanti JL, Davidson AD (2009) Identification of amino acids in the dengue virus type 2 envelope glycoprotein critical to virus infectivity. J Gen Virol 90: 2457–2461. 19587132 10.1099/vir.0.011486-0

[33] Zheng A, Umashankar M, Kielian M (2010) In vitro and in vivo studies identify important features of dengue virus pr-E protein interactions. PLoS Pathog 6: e1001157. 20975939 10.1371/journal.ppat.1001157 PMC2958806

[34] Thitithanyanont A, Engering A, Ekchariyawat P, Wiboon-ut S, Limsalakpetch A, et al (2007) High susceptibility of human dendritic cells to avian influenza H5N1 virus infection and protection by IFN-alpha and TLR ligands. J Immunol 179: 5220–5227. 17911607 10.4049/jimmunol.179.8.5220

[35] Westcott MM, Ahmed M, Smedberg JR, Rajani KR, Hiltbold EM, et al (2013) Preservation of dendritic cell function during vesicular stomatitis virus infection reflects both intrinsic and acquired mechanisms of resistance to suppression of host gene expression by viral M protein. J Virol 87: 11730–11740. 23986580 10.1128/JVI.00680-13 PMC3807369

[36] Mukhopadhyay S, Kuhn RJ, Rossmann MG (2005) A structural perspective of the flavivirus life cycle. Nat Rev Microbiol 3: 13–22. 15608696 10.1038/nrmicro1067

[37] Macia E, Ehrlich M, Massol R, Boucrot E, Brunner C, et al (2006) Dynasore, a cell-permeable inhibitor of dynamin. Dev Cell 10: 839–850. 16740485 10.1016/j.devcel.2006.04.002

[38] Wang LH, Rothberg KG, Anderson RG (1993) Mis-assembly of clathrin lattices on endosomes reveals a regulatory switch for coated pit formation. J Cell Biol 123: 1107–1117. 8245121 10.1083/jcb.123.5.1107 PMC2119875

[39] Achuthan A, Masendycz P, Lopez JA, Nguyen T, James DE, et al (2008) Regulation of the endosomal SNARE protein syntaxin 7 by colony-stimulating factor 1 in macrophages. Mol Cell Biol 28: 6149–6159. 18710945 10.1128/MCB.00220-08 PMC2577439

[40] Haspot F, Lavault A, Sinzger C, Laib Sampaio K, Stierhof YD, et al (2012) Human cytomegalovirus entry into dendritic cells occurs via a macropinocytosis-like pathway in a pH-independent and cholesterol-dependent manner. PLoS ONE 7: e34795. 22496863 10.1371/journal.pone.0034795 PMC3322158

[41] Russo C, Cornella-Taracido I, Galli-Stampino L, Jain R, Harrington E, et al (2011) Small molecule Toll-like receptor 7 agonists localize to the MHC class II loading compartment of human plasmacytoid dendritic cells. Blood 117: 5683–5691. 21487111 10.1182/blood-2010-12-328138

[42] Bershadsky A, Chausovsky A, Becker E, Lyubimova A, Geiger B (1996) Involvement of microtubules in the control of adhesion-dependent signal transduction. Curr Biol 6: 1279–1289. 8939572 10.1016/s0960-9822(02)70714-8

[43] Eligini S, Songia P, Cavalca V, Crisci M, Tremoli E, et al (2012) Cytoskeletal architecture regulates cyclooxygenase-2 in human endothelial cells: autocrine modulation by prostacyclin. J Cell Physiol 227: 3847–3856. 22495438 10.1002/jcp.24097

[44] Young KG, Thurston SF, Copeland S, Smallwood C, Copeland JW (2008) INF1 is a novel microtubule-associated formin. Mol Biol Cell 19: 5168–5180. 18815276 10.1091/mbc.E08-05-0469 PMC2592679

[45] Mateo R, Nagamine CM, Spagnolo J, Mendez E, Rahe M, et al (2013) Inhibition of cellular autophagy deranges dengue virion maturation. J Virol 87: 1312–1321. 23175363 10.1128/JVI.02177-12 PMC3554187

[46] Bhatt S, Gething PW, Brady OJ, Messina JP, Farlow AW, et al (2013) The global distribution and burden of dengue. Nature 496: 504–507. 23563266 10.1038/nature12060 PMC3651993

[47] Whitehorn J, Simmons CP (2011) The pathogenesis of dengue. Vaccine 29: 7221–7228. 21781999 10.1016/j.vaccine.2011.07.022

[48] Gandini M, Gras C, Azeredo EL, Pinto LM, Smith N, et al (2013) Dengue virus activates membrane TRAIL relocalization and IFN-alpha production by human plasmacytoid dendritic cells in vitro and in vivo. PLoS Negl Trop Dis 7: e2257. 23755314 10.1371/journal.pntd.0002257 PMC3675005

[49] Pichyangkul S, Endy TP, Kalayanarooj S, Nisalak A, Yongvanitchit K, et al (2003) A blunted blood plasmacytoid dendritic cell response to an acute systemic viral infection is associated with increased disease severity. J Immunol 171: 5571–5578. 14607965 10.4049/jimmunol.171.10.5571

[50] Rodriguez-Madoz JR, Bernal-Rubio D, Kaminski D, Boyd K, Fernandez-Sesma A (2010) Dengue virus inhibits the production of type I interferon in primary human dendritic cells. J Virol 84: 4845–4850. 20164230 10.1128/JVI.02514-09 PMC2863727

[51] Sun P, Fernandez S, Marovich MA, Palmer DR, Celluzzi CM, et al (2009) Functional characterization of ex vivo blood myeloid and plasmacytoid dendritic cells after infection with dengue virus. Virology 383: 207–215. 19013627 10.1016/j.virol.2008.10.022

[52] Yu IM, Zhang W, Holdaway HA, Li L, Kostyuchenko VA, et al (2008) Structure of the immature dengue virus at low pH primes proteolytic maturation. Science 319: 1834–1837. 18369148 10.1126/science.1153264

[53] Moesker B, Rodenhuis-Zybert IA, Meijerhof T, Wilschut J, Smit JM (2010) Characterization of the functional requirements of West Nile virus membrane fusion. J Gen Virol 91: 389–393. 19828760 10.1099/vir.0.015255-0

[54] Yu IM, Holdaway HA, Chipman PR, Kuhn RJ, Rossmann MG, et al (2009) Association of the pr peptides with dengue virus at acidic pH blocks membrane fusion. J Virol 83: 12101–12107. 19759134 10.1128/JVI.01637-09 PMC2786737

[55] Blasius AL, Beutler B (2010) Intracellular toll-like receptors. Immunity 32: 305–315. 20346772 10.1016/j.immuni.2010.03.012

[56] Cherrier MV, Kaufmann B, Nybakken GE, Lok SM, Warren JT, et al (2009) Structural basis for the preferential recognition of immature flaviviruses by a fusion-loop antibody. Embo J 28: 3269–3276. 19713934 10.1038/emboj.2009.245 PMC2771083

[57] Plevka P, Battisti AJ, Junjhon J, Winkler DC, Holdaway HA, et al (2011) Maturation of flaviviruses starts from one or more icosahedrally independent nucleation centres. EMBO Rep 12: 602–606. 21566648 10.1038/embor.2011.75 PMC3128282

[58] Dejnirattisai W, Jumnainsong A, Onsirisakul N, Fitton P, Vasanawathana S, et al (2010) Cross-reacting antibodies enhance dengue virus infection in humans. Science 328: 745–748. 20448183 10.1126/science.1185181 PMC3837288

[59] Beltramello M, Williams KL, Simmons CP, Macagno A, Simonelli L, et al (2010) The human immune response to Dengue virus is dominated by highly cross-reactive antibodies endowed with neutralizing and enhancing activity. Cell Host Microbe 8: 271–283. 20833378 10.1016/j.chom.2010.08.007 PMC3884547

[60] Luo YY, Feng JJ, Zhou JM, Yu ZZ, Fang DY, et al (2013) Identification of a novel infection-enhancing epitope on dengue prM using a dengue cross-reacting monoclonal antibody. BMC Microbiol 13: 194. 23987307 10.1186/1471-2180-13-194 PMC3765915

[61] Galloway SE, Reed ML, Russell CJ, Steinhauer DA (2013) Influenza HA subtypes demonstrate divergent phenotypes for cleavage activation and pH of fusion: implications for host range and adaptation. PLoS Pathog 9: e1003151. 23459660 10.1371/journal.ppat.1003151 PMC3573126

[62] Matczuk AK, Kunec D, Veit M (2013) Co-translational processing of glycoprotein 3 from equine arteritis virus: N-glycosylation adjacent to the signal peptide prevents cleavage. J Biol Chem 288: 35396–35405. 24142700 10.1074/jbc.M113.505420 PMC3853287

[63] Fujinami RS, Oldstone MB (1981) Failure to cleave measles virus fusion protein in lymphoid cells. J Exp Med 154: 1489–1499. 6895382 10.1084/jem.154.5.1489 PMC2186533

[64] Steinhauer DA (1999) Role of hemagglutinin cleavage for the pathogenicity of influenza virus. Virology 258: 1–20. 10329563 10.1006/viro.1999.9716

[65] Zhong J, Gastaminza P, Cheng G, Kapadia S, Kato T, et al (2005) Robust hepatitis C virus infection in vitro. Proc Natl Acad Sci U S A 102: 9294–9299. 15939869 10.1073/pnas.0503596102 PMC1166622

[66] Pryor MJ, Carr JM, Hocking H, Davidson AD, Li P, et al (2001) Replication of dengue virus type 2 in human monocyte-derived macrophages: comparisons of isolates and recombinant viruses with substitutions at amino acid 390 in the envelope glycoprotein. Am J Trop Med Hyg 65: 427–434. 11716094 10.4269/ajtmh.2001.65.427

[67] Pierson TC, Diamond MS, Ahmed AA, Valentine LE, Davis CW, et al (2005) An infectious West Nile virus that expresses a GFP reporter gene. Virology 334: 28–40. 15749120 10.1016/j.virol.2005.01.021

[68] Ostertag D, Hoblitzell-Ostertag TM, Perrault J (2007) Overproduction of double-stranded RNA in vesicular stomatitis virus-infected cells activates a constitutive cell-type-specific antiviral response. J Virol 81: 503–513. 17065213 10.1128/JVI.01218-06 PMC1797476

[69] Le Goffic R, Bouguyon E, Chevalier C, Vidic J, Da Costa B, et al (2010) Influenza A virus protein PB1-F2 exacerbates IFN-beta expression of human respiratory epithelial cells. J Immunol 185: 4812–4823. 20844191 10.4049/jimmunol.0903952

[70] Sumpter R, Loo YM, Foy E, Li K, Yoneyama M, et al (2005) Regulating intracellular antiviral defense and permissiveness to hepatitis C virus RNA replication through a cellular RNA helicase, RIG-I. J Virol 79: 2689–2699. 15708988 10.1128/JVI.79.5.2689-2699.2005 PMC548482

[71] Meertens L, Carnec X, Lecoin MP, Ramdasi R, Guivel-Benhassine F, et al (2012) The TIM and TAM families of phosphatidylserine receptors mediate dengue virus entry. Cell Host Microbe 12: 544–557. 23084921 10.1016/j.chom.2012.08.009 PMC3572209

[72] Dreux M, Dao Thi VL, Fresquet J, Guerin M, Julia Z, et al (2009) Receptor complementation and mutagenesis reveal SR-BI as an essential HCV entry factor and functionally imply its intra- and extra-cellular domains. PLoS Pathog 5: e1000310. 19229312 10.1371/journal.ppat.1000310 PMC2636890

[73] Pryor MJ, Azzola L, Wright PJ, Davidson AD (2004) Histidine 39 in the dengue virus type 2 M protein has an important role in virus assembly. J Gen Virol 85: 3627–3636. 15557235 10.1099/vir.0.80283-0

