# Supplementary figures and images for: Sensing of Immature Particles Produced by Dengue Virus Infected Cells Induces an Antiviral Response by Plasmacytoid Dendritic Cells
Source: PLoS Pathog. 2014 Oct 23;10(10):e1004434. doi: 10.1371/journal.ppat.1004434 (PMC4207819; doi:10.1371/journal.ppat.1004434)

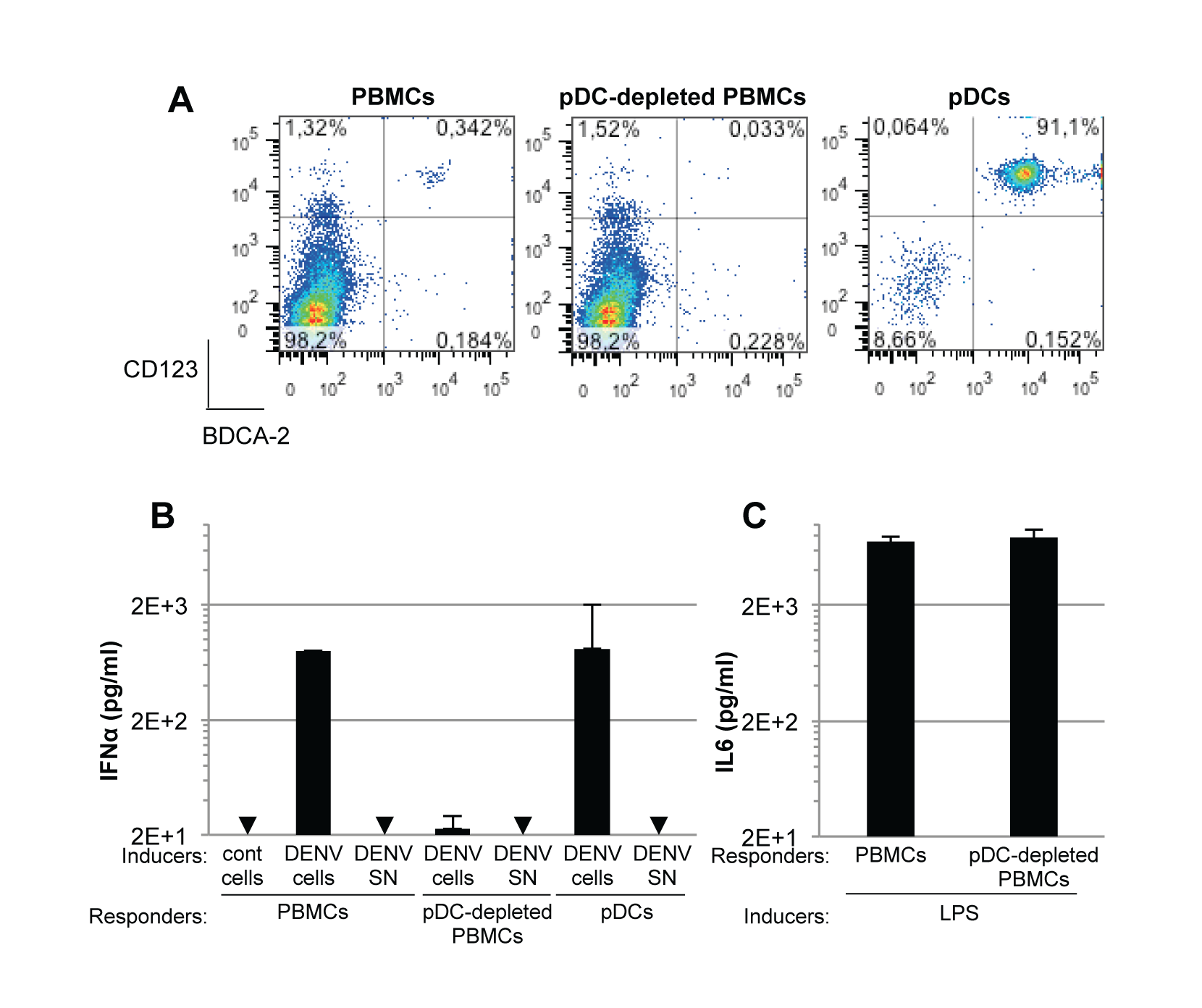

Supplement: Figure S1 — pDCs robustly produce IFNα in response to DENV infected cells, related to Figure 1 . pDC depletion or enrichment was performed using an anti-BDCA-4 antibody. (A) Representative FACS analysis of pDC depletion and isolation from PBMCs using the pDC selective markers, CD123 and BDCA-2. (B) Quantification of IFNα in the supernatants of 105 PBMCs, 105 pDC depleted-PBMCs and 103 isolated pDCs (Responders) co-cultured with DENV infected BHK-21 cells (DENV cells) or treated with 100 µl of supernatants from the latter cells (DENV SN), in a 200 µl final volume. Viral titers in the DENV SN were ≈0.75×10^6^ foci forming units (ffu)/ml. Uninfected BHK-21 cells are referred to as control (cont) cells. Arrows indicate results below the detection threshold of the IFNα ELISA (i.e., 12.5 pg/ml). Results are representative of 3 independent experiments in triplicate. Error bars represent the means ± SD. (C) Parallel quantification of IL6 in the supernatants of PBMCs or pDC-depleted PBMCs (Responders) triggered by incubation with LPS (10 µg/mL for 20 hours). Results are representative of 3 independent experiments in triplicates. Error bars represent the means ± SD. (TIF) [file ppat.1004434.s001.tif]

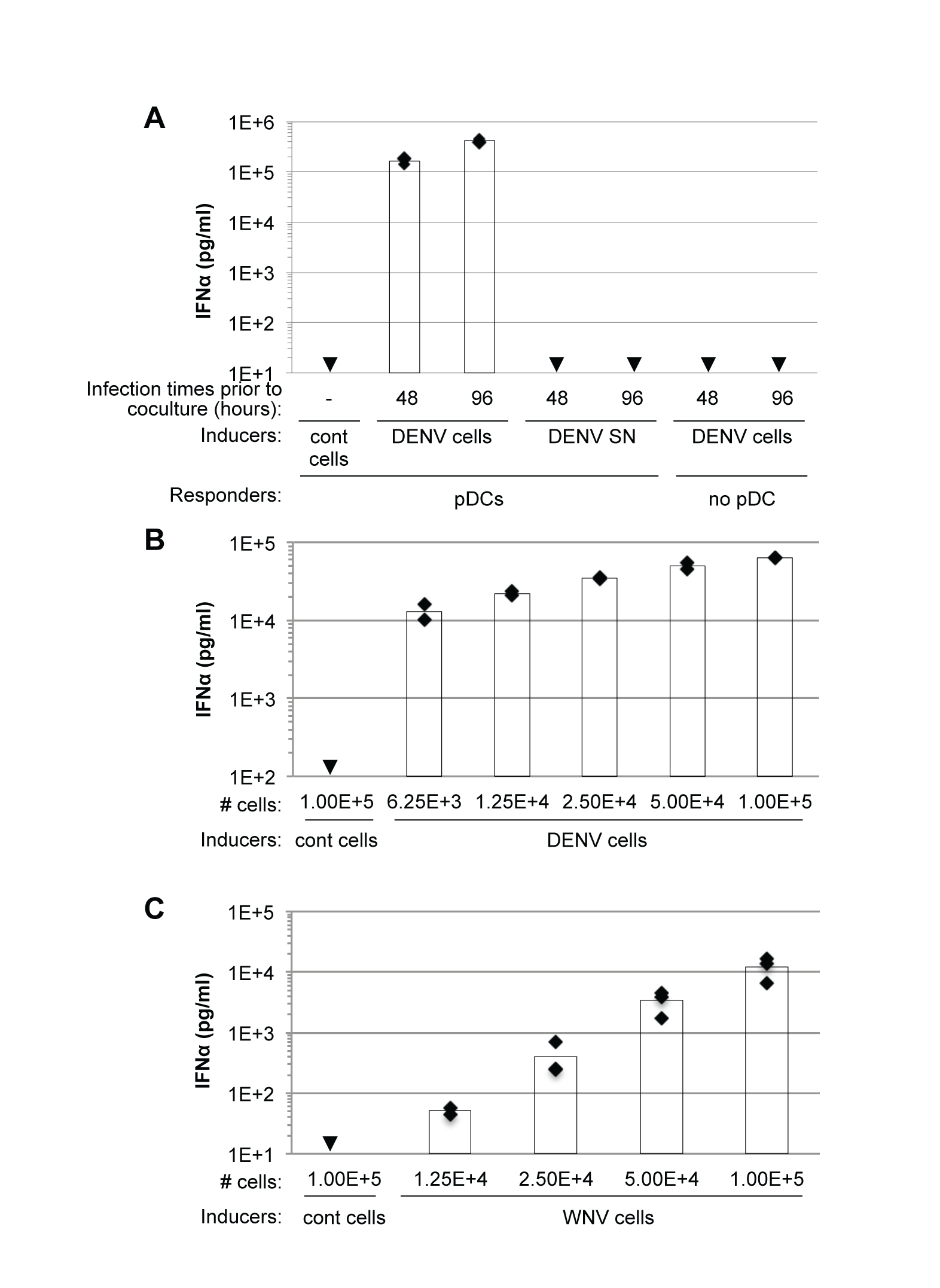

Supplement: Figure S2 — Effect of infection time duration and of varying the number of infected cells on pDC IFNα secretion, related to Figure 1 . (A) Quantification of IFNα in the supernatants of pDCs co-cultured with DENV cells or incubated with 100 µl of their supernatants (DENV SN) in a 200 µl final volume. Cells were infected at a MOI of 0.2 either 48 or 96 hours prior to co-culture, as indicated. Viral titers of the DENV SN were ≈105 and 4×10^5^ ffu/ml at 48 and 96 hours, respectively. Results of 2 independent experiments in duplicates. Diamonds and histograms represent individual repeats and means, respectively. (B–C) Quantification of IFNα in supernatants of pDCs co-cultured with varying numbers of DENV infected cells (DENV cells) (B) or WNV infected cells (WNV cells) (C), Results of 2 (B) and 3 (C) independent experiments in duplicates. Diamonds and histograms represent individual repeats and means, respectively. Uninfected cells are referred to as control (cont) cells. Arrows indicate results below the limit of detection of the IFNα ELISA (i.e., 12.5 pg/ml). # cells; number of infected cells. (TIF) [file ppat.1004434.s002.tif]

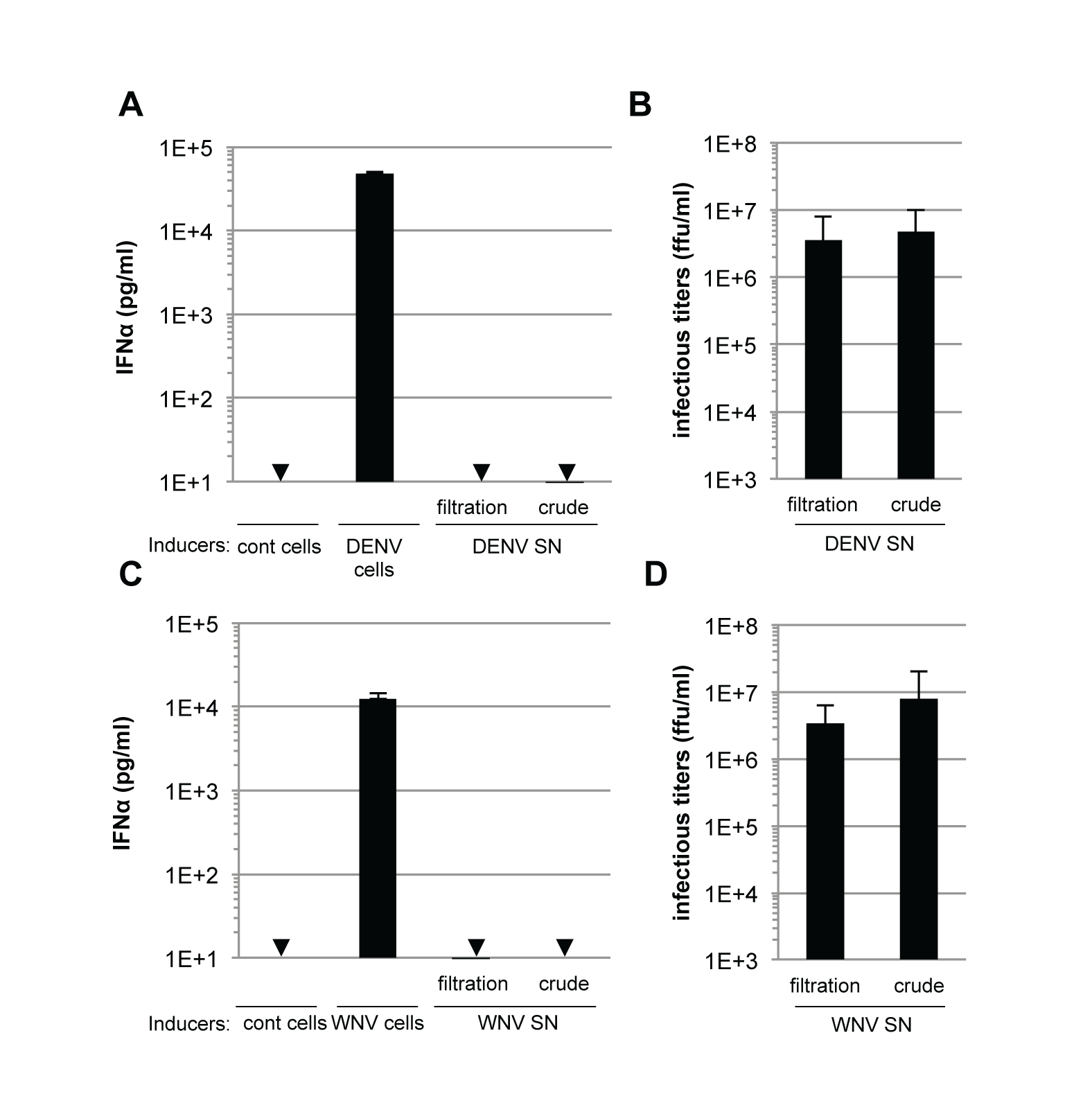

Supplement: Figure S3 — Cell-free virus containing supernatants fail to trigger IFNα by pDCs. Quantification of IFNα in supernatants of pDCs co-cultured with cells infected by DENV cells (A) or by WNV (C) or inoculated with 100 µl of their supernatants (SN) that were filtrated using 0.45 µm filters (filtration) or not (crude). Arrows indicate results below the limit of detection of the IFNα ELISA (i.e., 12.5 pg/ml). (B–D) Parallel determination of infectious viral titers of DENV SN (B) and WNV SN (D). Results are representative of 3 independent experiments in triplicates. Error bars represent the means ± SD. (TIF) [file ppat.1004434.s003.tif]

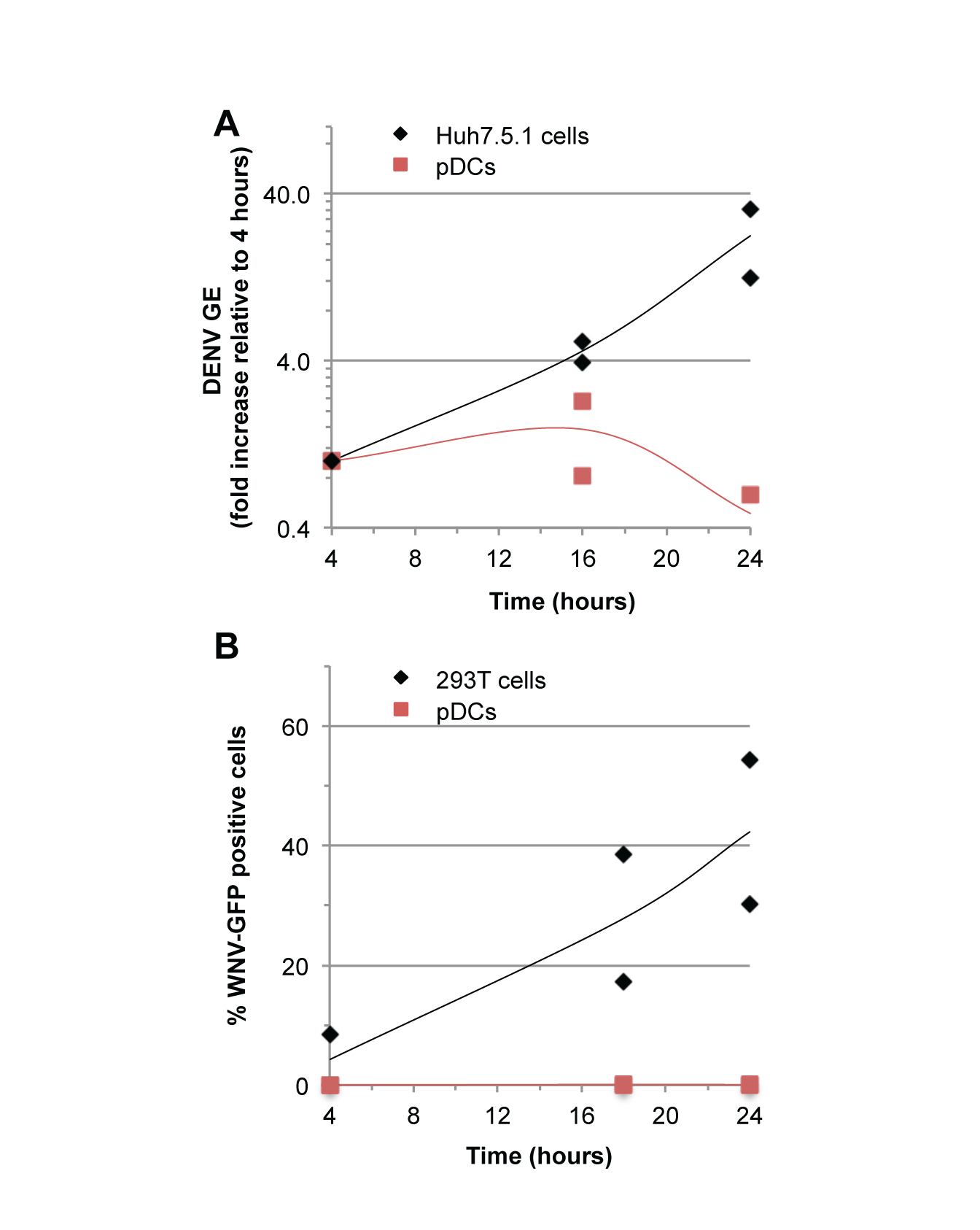

Supplement: Figure S4 — pDCs are not permissive to DENV and WNV infections. (A) Time course analysis of intracellular DENV RNA levels by RT-qPCR. Results are expressed as fold increase relative to the detection at 4 hours post-inoculation with a MOI of 1 for Huh7.5.1 and MOI of 3 for pDCs. Results are expressed as the fold increase of DENV genome equivalents (GE) relative to the 4 hour time point. Results of 2 independent experiments in duplicates. Diamonds represent individual repeats. (B) Time course analysis of the percentage of WNV-GFP positive cells after inoculation with WNV-GFP at a MOI of 4 for 293T cells and MOI of 12 for pDCs. Results are quantified by FACS. Results of 2 independent experiments in duplicates. Diamonds represent individual repeats. (TIF) [file ppat.1004434.s004.tif]

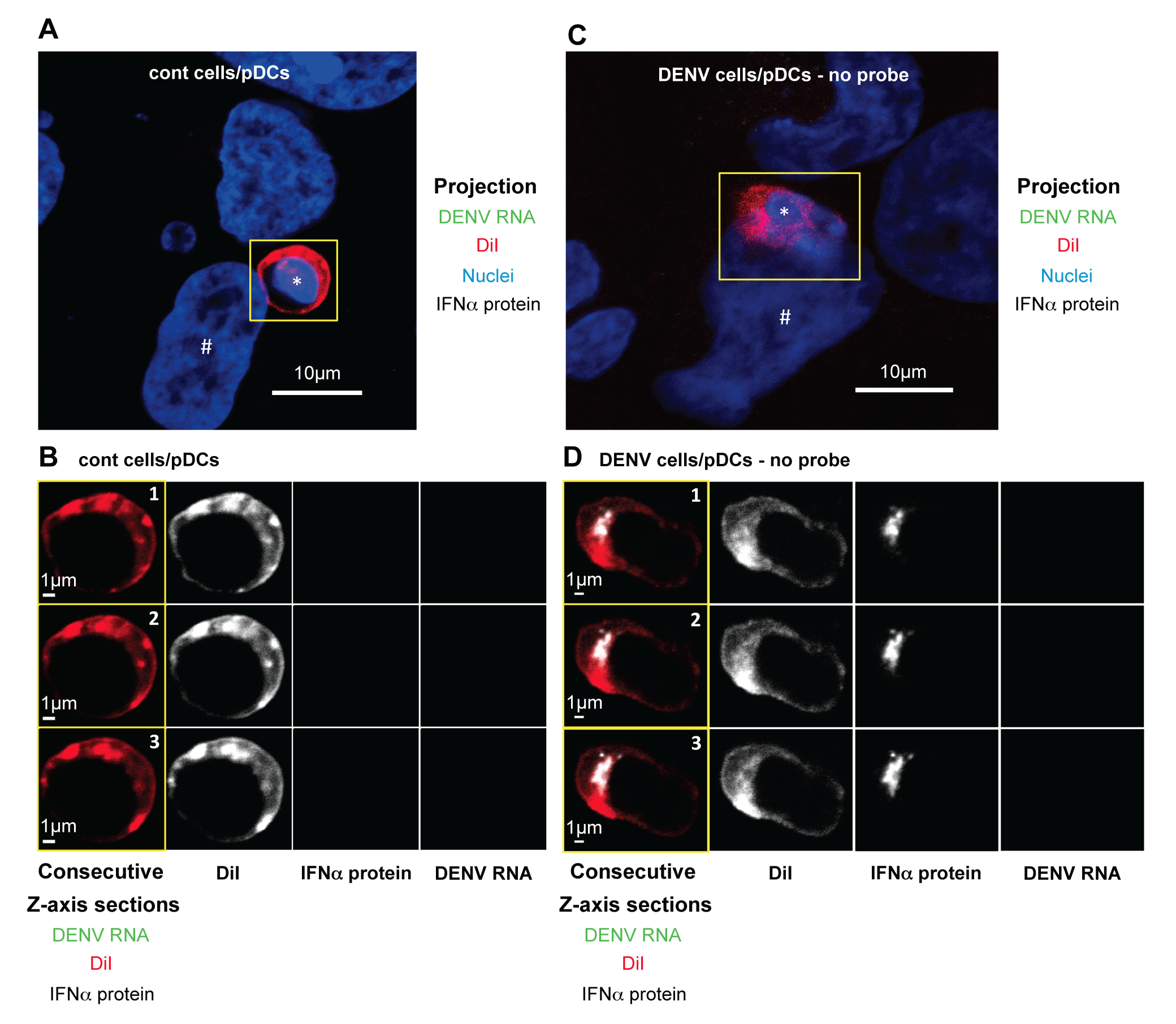

Supplement: Figure S5 — Specificity of the detection of DENV RNA transferred from infected cells to pDCs by RNA FISH assays, related to Figure 5 . Confocal microscopy analysis of DENV RNA detected by FISH in co-cultures of pDCs with uninfected Huh-7.5.1 cells (Cont cells/pDCs) (A–B) and DENV infected Huh-7.5.1 cells with omission of DENV-specific probe (DENV cells/pDCs – no probe) (C–D). Cells were co-cultured for 5 hours, followed by the same procedure of hybridization and immunostaining as described in Figure 5A. (A–C) Projections of confocal microscopy analysis with DENV RNA (green), DiI-stained pDC (red), IFNα protein (white) and nuclei (blue). Star mark (*) indicates the pDC and hash mark (#) indicates the Huh7.5.1 cell. (B–D) Consecutive Z-axis sections with magnification of yellow-boxed pDC, shown in the corresponding panels (A) and (C). Left panels, combined detections of DENV RNA (green), DiI-stained pDC (red), IFNα protein (white). Their individual detections are displayed on the right panels as indicated. Similar results were obtained in 3 independent experiments. (TIF) [file ppat.1004434.s005.tif]

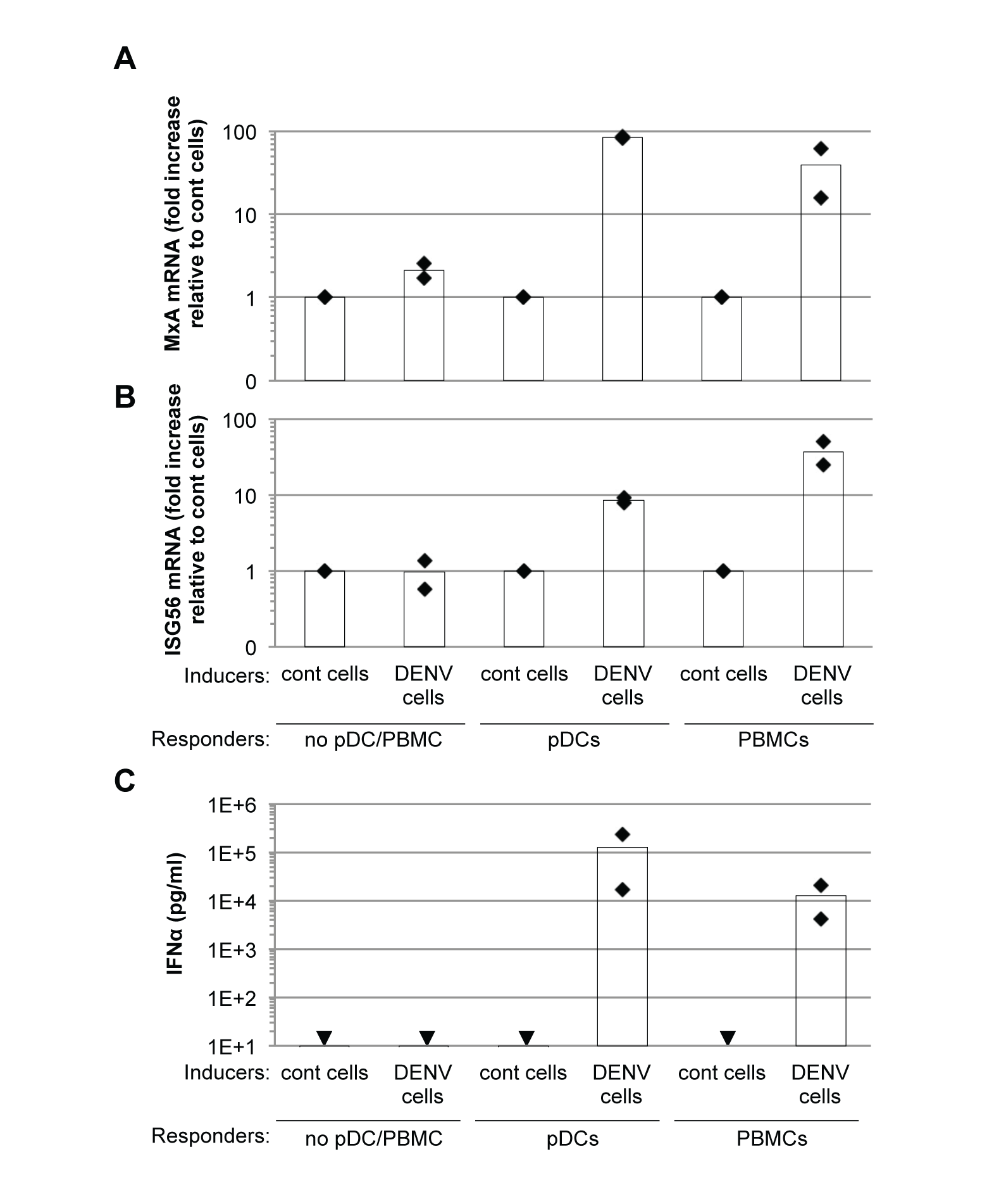

Supplement: Figure S6 — Induction of ISG expressions by pDCs or PBMCs co-cultured with DENV infected cells. Quantification of intracellular MxA (A) and ISG56 (B) mRNA levels of pDCs or PBMCs co-cultured or not with DENV infected Huh7.5.1 cells (DENV cells). Results are expressed as fold increase relative to corresponding co-culture with uninfected Huh7.5.1 (cont) cells. (C) Parallel quantification of IFNα in supernatants of pDCs or PBMCs co-cultured or not with DENV infected Huh7.5.1 cells (DENV cells). Results of 2 independent experiments in duplicates. Diamonds and histograms represent individual repeats and means, respectively. Arrows indicate results below the limit of detection of the IFNα ELISA (i.e., 12.5 pg/ml). (TIF) [file ppat.1004434.s006.tif]

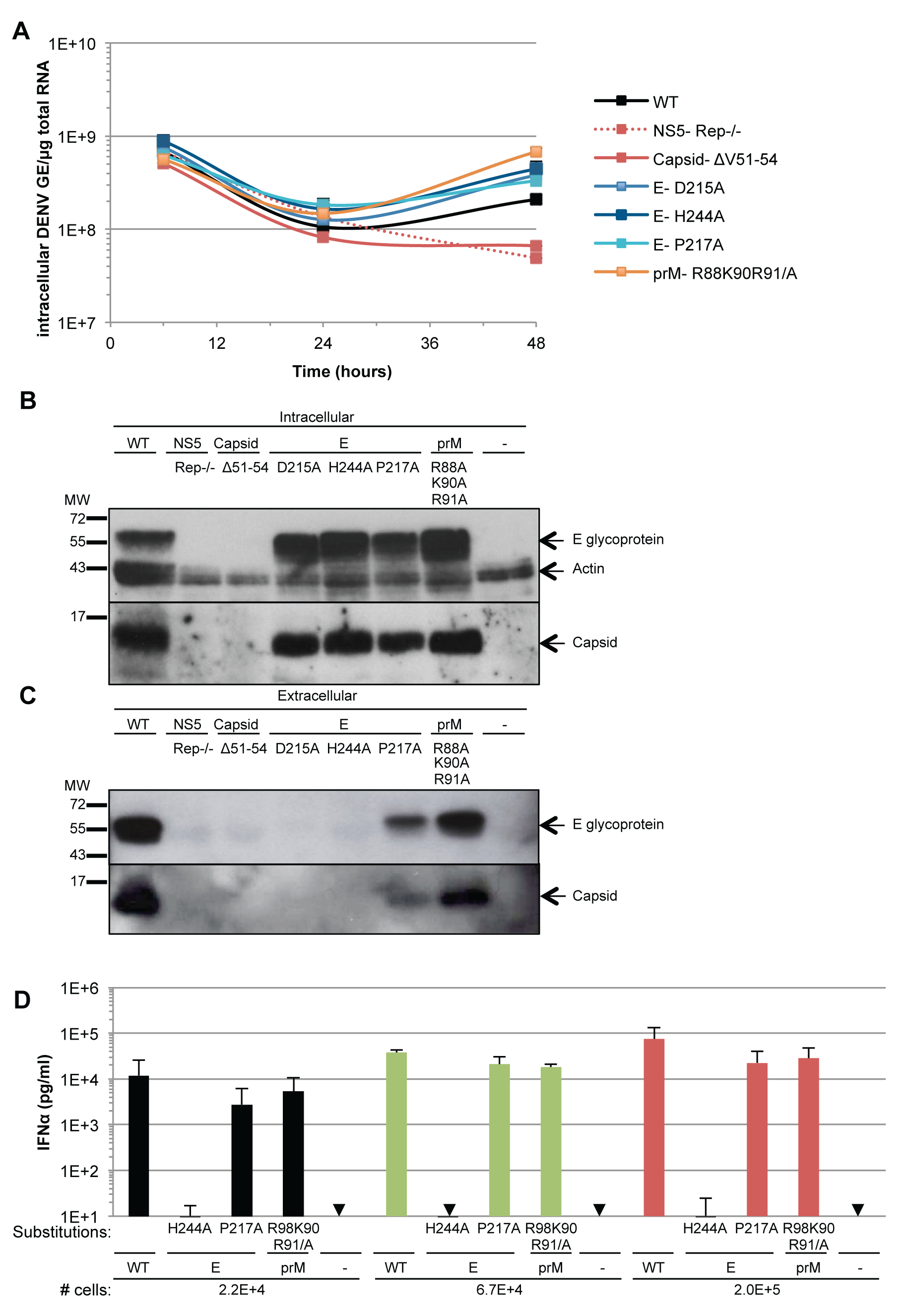

Supplement: Figure S7 — Impact of mutations in DENV NS5, capsid and E and prM on intracellular and extracellular levels of viral proteins, related to Figures 6 and 9 . (A) The impact of mutations on levels of intracellular DENV RNAs at the indicated time post-transfection. Results are quantified by RT-qPCR and expressed as DENV genome equivalent (GE)/µg total RNA. (B–C) Representative Western blot analyses of intracellular (B) and extracellular (C) DENV E and capsid protein levels. Detection of the actin protein was used as a loading control. MW; molecular weight markers in kDa. Results are representative of 3 independent experiments. (D) Effect of varying numbers of donor cells on pDC IFNα secretion. Quantification of IFNα in supernatants of pDCs co-cultured with varying numbers of cells harboring the WT and mutant DENV genomes (means ± SD, n = 3). Cells harvested at 24 hours post-transfection were co-cultured with pDCs for 20 hours. Uninfected cells are referred to as control (-) cells. Arrows indicate results below the limit of detection of the IFNα ELISA (i.e., 12.5 pg/ml). # cells; number of infected cells. (TIF) [file ppat.1004434.s007.tif]

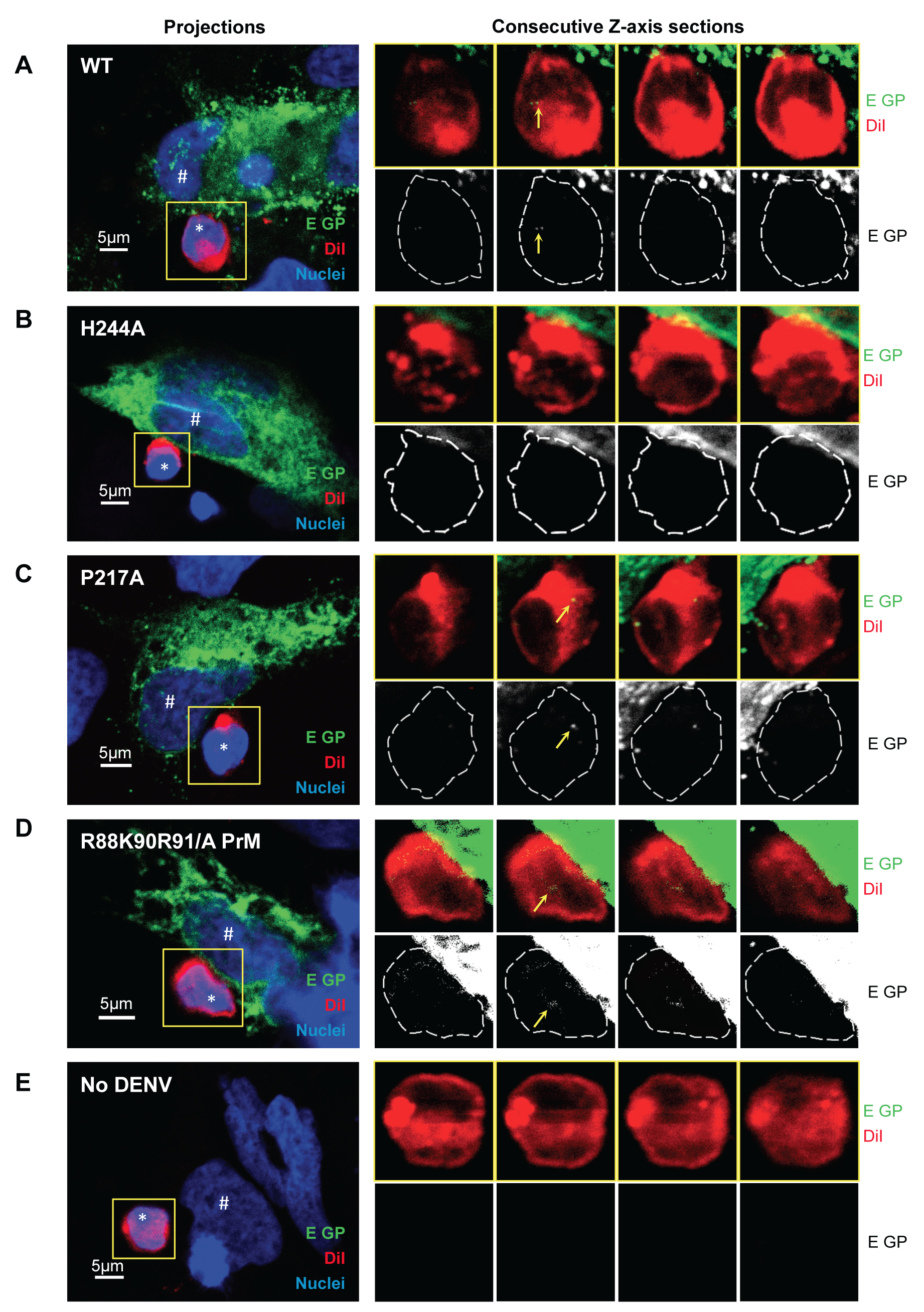

Supplement: Figure S8 — Transfer of DENV E glycoproteins from cells harboring the WT and mutant DENV genomes to co-cultured pDCs, related to Figures 6 and 9 . Left panels, representative projections of confocal microscopy analysis of DENV E glycoproteins (E GP, green) detected by immunostaining in co-cultures of pDCs stained by DiI dye (red) and cells harboring the WT genome (A), or genome with mutations in E, i.e., substitutions H244A (B) and P217A (C), with mutations in prM, i.e., substitutions R88A, K90A and R91A (D) or cells devoid of DENV genome, as control (E); nuclei (blue). Right panels, consecutive Z-axis sections with magnification of yellow-boxed pDC, shown in the corresponding left panels. Cell contours on E GP panels are labeled with dotted lines surrounding DiI staining. Yellow-arrows; E GP dots inside pDC. Star mark (*) and hash mark (#) indicates the pDC and the Huh7.5.1 cell, respectively. Similar results were obtained in 3 independent experiments and summary table of the statistical analysis is displayed in Figure 6E. (TIF) [file ppat.1004434.s008.tif]

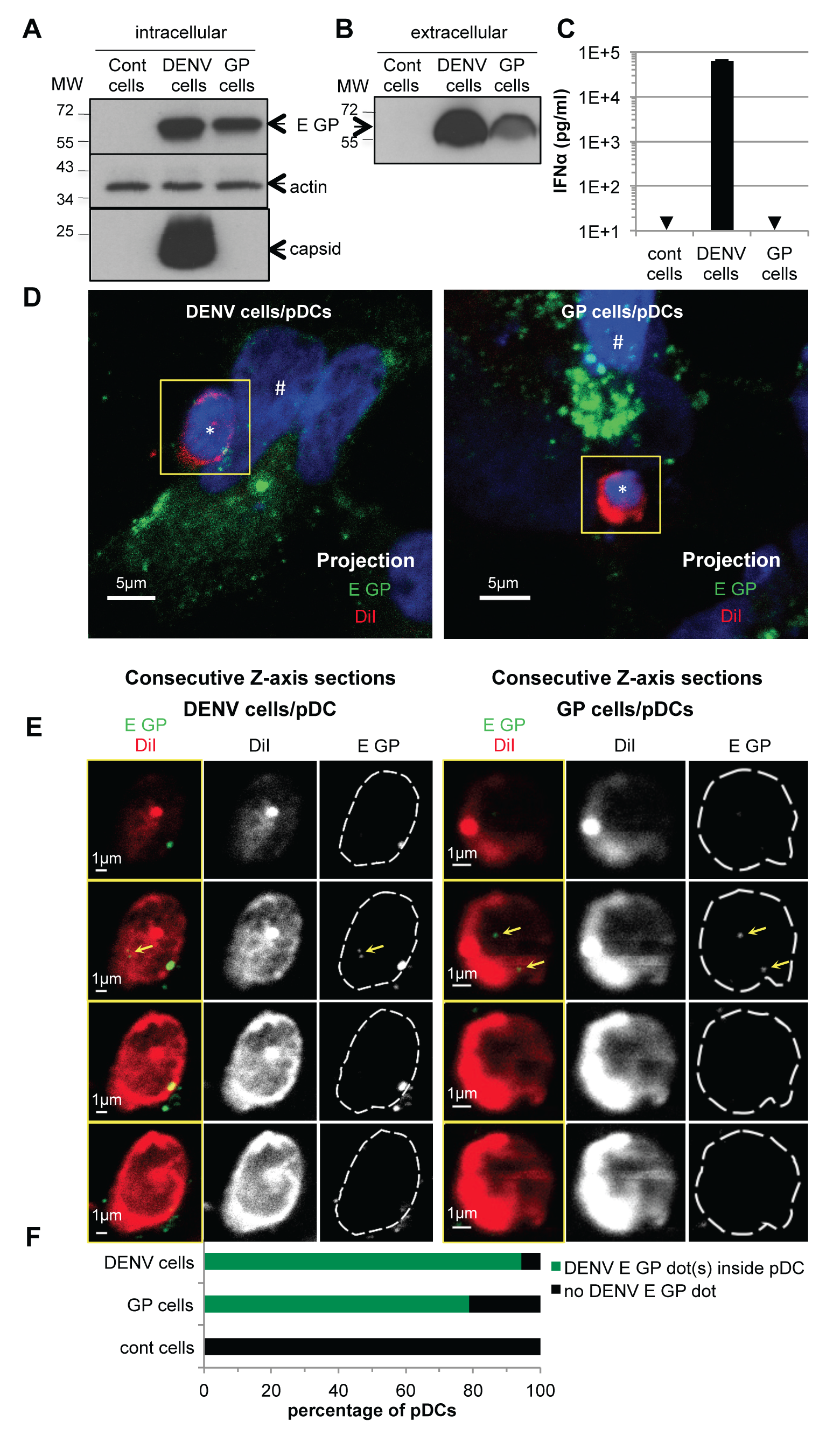

Supplement: Figure S9 — Individual expression of DENV surface proteins ( i.e. , E GP and prM) and their transmission into the pDCs are not sufficient to trigger IFNα production. Representative Western blot analyses of intracellular DENV E and capsid protein levels (A) and extracellular DENV E protein levels (B) with cells expressing only DENV glycoproteins (GP cells) as compared to DENV infected cells (DENV cells) and uninfected parental cells (Cont cells). Detection of the actin protein used as a loading control, MW; molecular weight markers in kDa. Results are representative of 3 independent experiments. (C) Quantification of IFNα in supernatants of pDCs co-cultured with cells expressing only DENV glycoproteins (GP cells) as compared to DENV infected cells (DENV cells) and uninfected parental cells (Cont cells). Arrows indicate results below the limit of detection of the IFNα ELISA (i.e. 12.5 pg/ml). Error bars represent the means ± SD, results are representative of 3 independent experiments. (D) Representative projections of confocal microscopy analysis of DENV E glycoproteins (E GP, green) detected by immunostaining in co-cultures of pDCs stained by DiI membrane dye (red) with cells expressing DENV glycoproteins (GP cells) as compared to DENV infected cells (DENV cells); nuclei (blue). Star mark (*): pDCs and hash mark (#): Huh7.5.1 cells. (E) Consecutive Z-axis sections with magnification of yellow-boxed pDC, shown in the corresponding upper panels. Cell contours on the E GP panels are labeled with dotted lines surrounding DiI staining. Yellow arrows; E GP dots inside pDC. Similar results were obtained in 3 independent experiments. (F) Results expressed as the percentages of DiI stained pDCs containing E GP dot(s). Similar results were obtained in 3 independent experiments and ≈20 pDCs, surrounded by at least one E GP positive cell, were observed per experimental condition. (TIF) [file ppat.1004434.s009.tif]

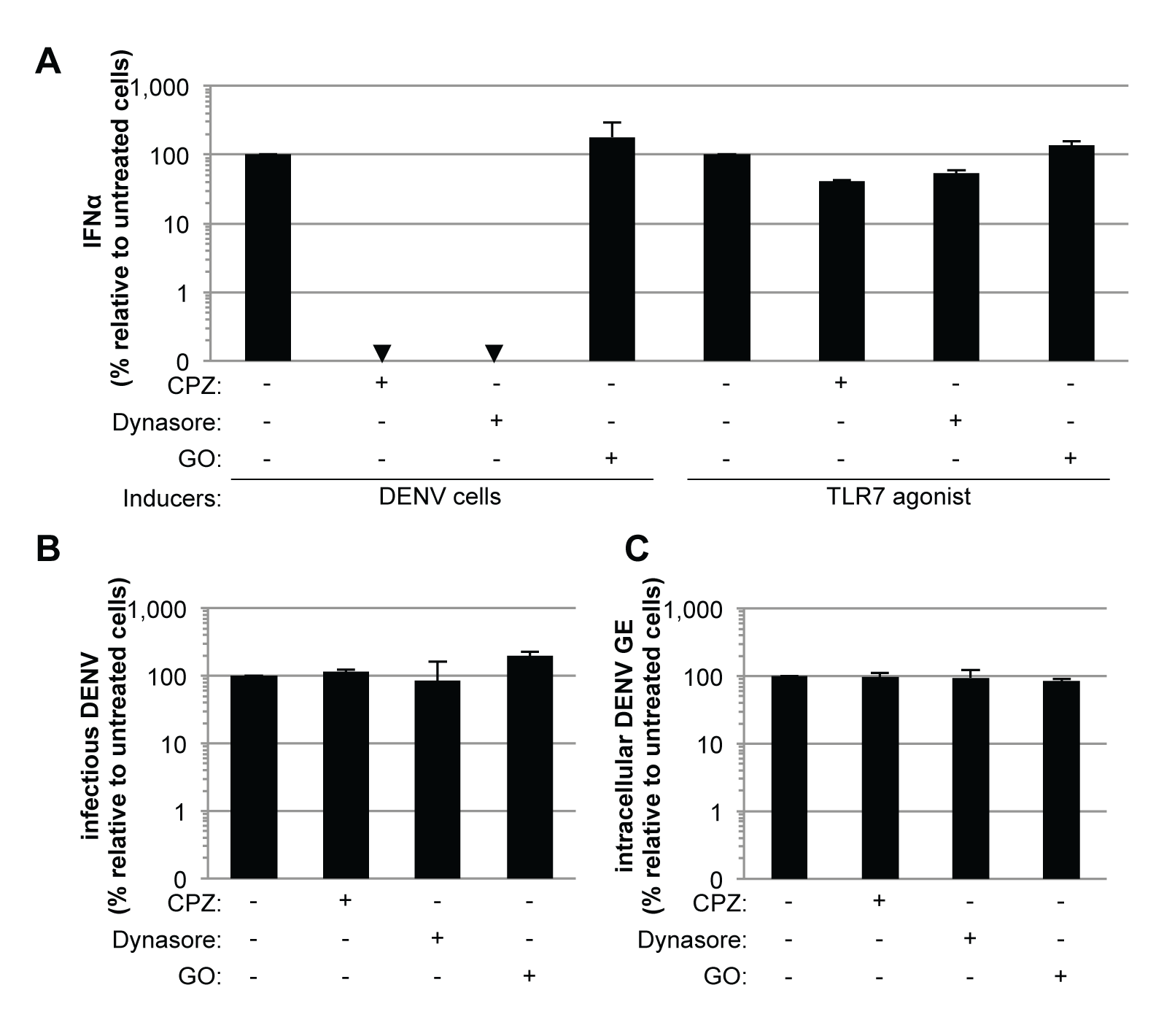

Supplement: Figure S10 — Impact of internalization inhibitors on IFNα production by pDCs co-cultured with DENV infected cells. Impact of inhibitors of clathrin-mediated endocytosis (chlorpromazine, CPZ, at 14 µM), of dynamin-dependent internalization (dynasore, at 100 µM) and macropinocytosis (Gö6983-PKC inhibitor, GO, at 5 µM) on pDC IFNα production triggered by DENV infected cells. (A) Quantification of IFNα in the supernatants of pDCs co-cultured with DENV infected Huh7.5.1 cells (DENV cells) or, as control, stimulated by TLR7 agonist the R848 (50 ng/mL), an imidazoquinoline known as a cell-permeable weak base that passively diffuses inside the pDCs. Results are expressed relative to IFNα produced in absence of inhibitor, set to 100 (means ± SD, n = 4). Arrows indicate results below the limit of detection of the IFNα ELISA (i.e., 12.5 pg/ml). Quantification of the levels of infectious virus production (B) and intracellular DENV genome equivalent (GE) (C) by Huh7.5.1 cells infected by DENV at MOI 3 for 48 hours (as for the co-culture in (A)) and then incubated, or not, with inhibitors, exactly as in (A) (i.e. incubation time and concentration). Results are expressed as percentages relative to untreated DENV cells (means ± SD, n = 4). (TIF) [file ppat.1004434.s010.tif]

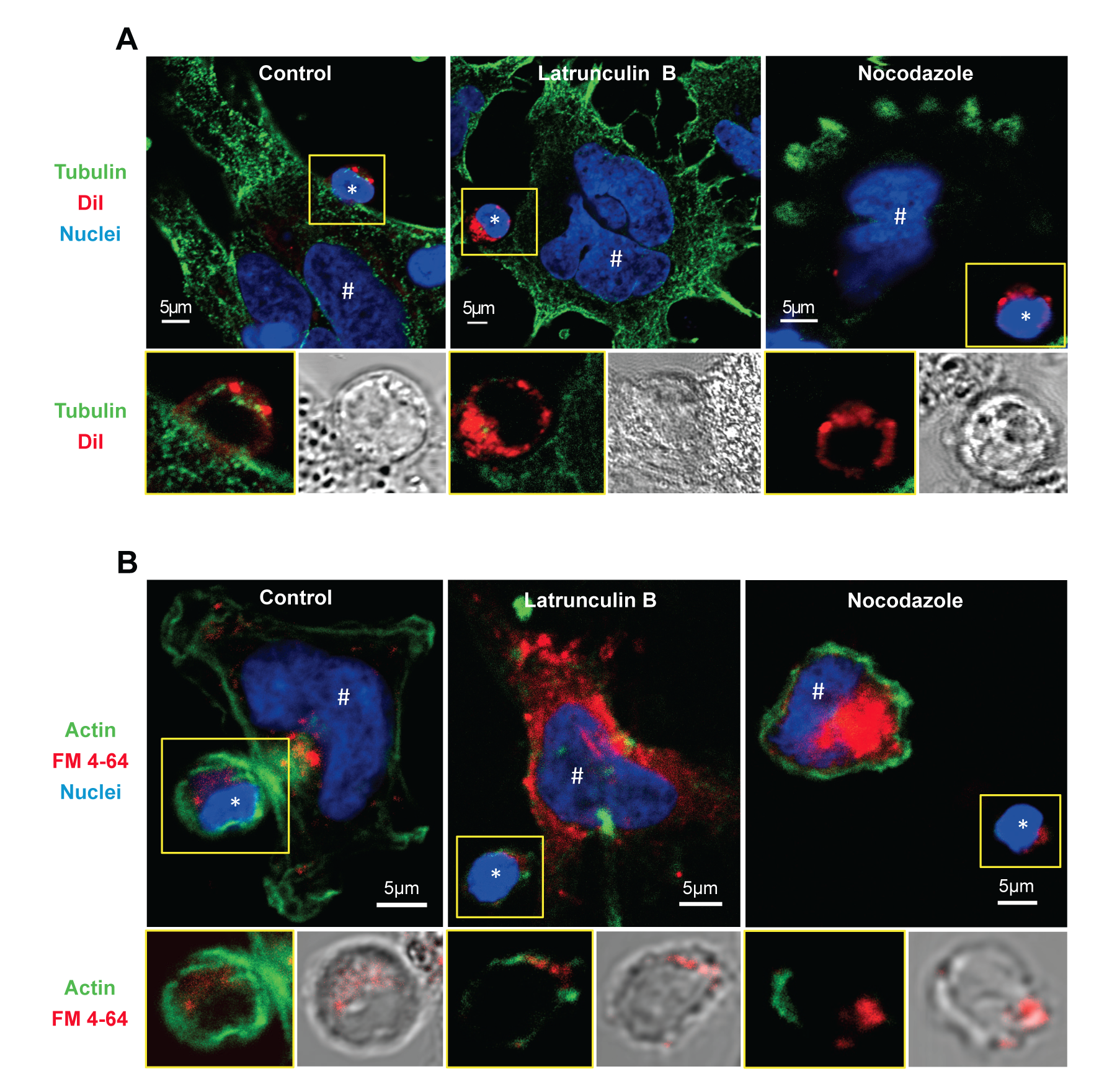

Supplement: Figure S11 — Impact of the cytoskeleton inhibitors on microtubule network and FM4-64 internalization, related to Figure 8 . (A) Imaging of immunostained α-tubulin in co-cultures of DiI-stained pDCs and DENV infected cells treated with cytoskeleton inhibitors, exactly as in Figure 8A. Star mark (*): pDCs and hash mark (#): Huh7.5.1 cells. Upper panels, confocal microscopy analysis of α-tubulin (green); DiI-stained pDC (red); nuclei (blue). Lower panels, magnification of yellow-boxed cell contact shown in the corresponding upper pictures with tubulin-DiI staining and phase contrast (left and right panels, respectively). Similar results were obtained in 2 independent experiments. (B) Imaging of the internalization of a lipophilic-dye, FM 4-64 (added for 15 min incubation at 37°C) in co-cultures of pDCs and DENV infected cells treated with cytoskeleton inhibitors, exactly as in Figure 8A. Upper panels, confocal microscopy analysis of actin (green); FM 4-64 (red); nuclei (blue). Lower panels, magnification of yellow boxes shown in the corresponding upper pictures, with actin-FM 4-64 and FM 4-64-phase contrast (left and right panels, respectively). (TIF) [file ppat.1004434.s011.tif]

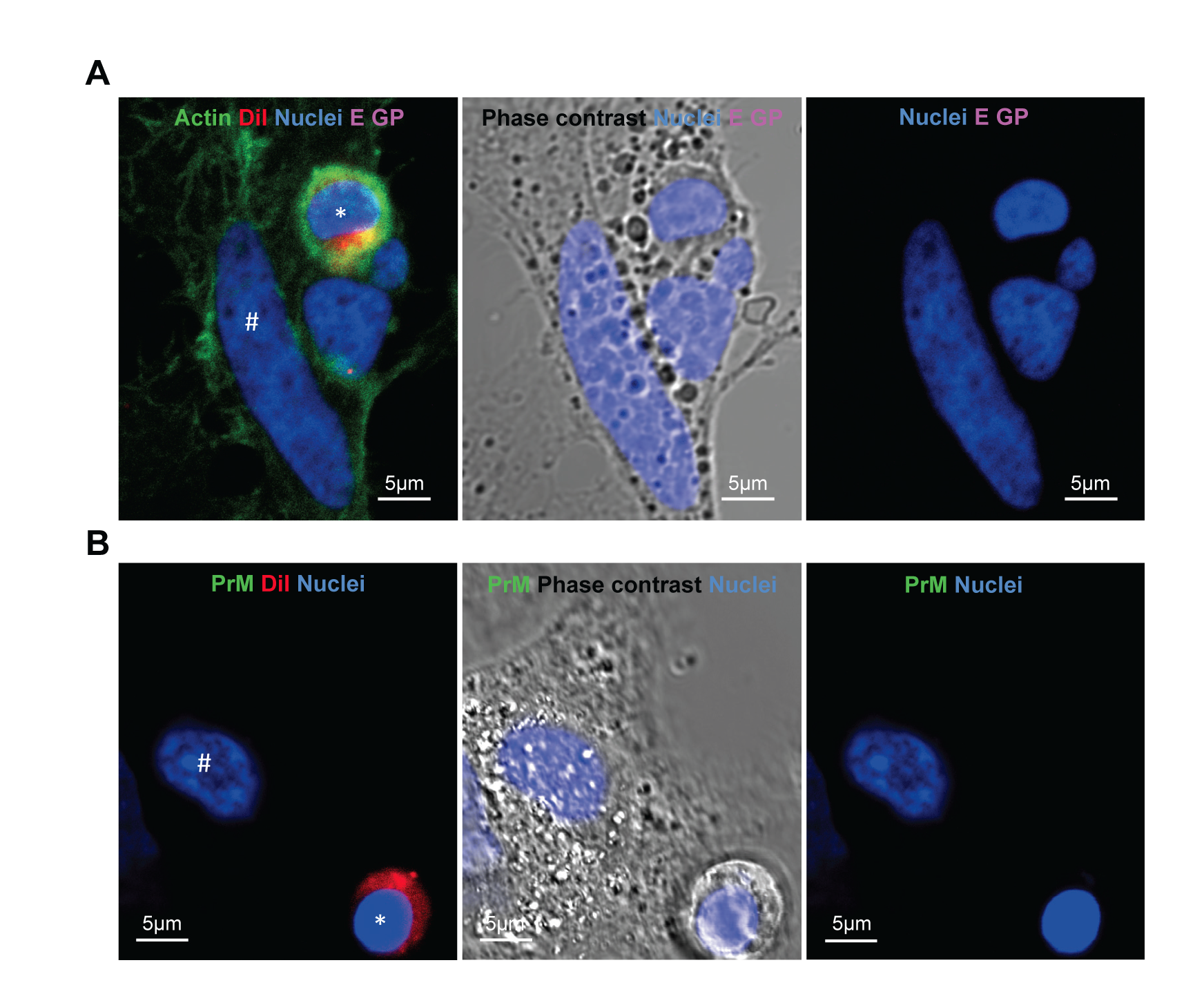

Supplement: Figure S12 — Specificity of the immuno-detection of DENV E and PrM clustering, related to Figure 7 . Absence of detection of E glycoprotein (E GP, purple) (A) and prM (green) (B) in co-cultures of DiI-stained pDCs (red) with uninfected Huh 7.5.1 cells, analyzed exactly as in Figure 7F–K and 7L–Q, respectively. Left panels, confocal analysis of DENV envelope proteins, E GP (purple), prM (green), DiI-stained pDCs (Red), actin detected by Alexa 488-conjugated phalloidin (green), when indicated, and nuclei (blue). Middle panels, confocal microscopy analysis of DENV envelope proteins and nuclei (blue) projected on the phase contrast imaging. Right panels, confocal microscopy analysis of DENV envelope proteins and nuclei (blue). Star mark (*): pDCs and hash mark (#): Huh7.5.1 cells. Similar results were obtained in 3 independent experiments. (TIF) [file ppat.1004434.s012.tif]

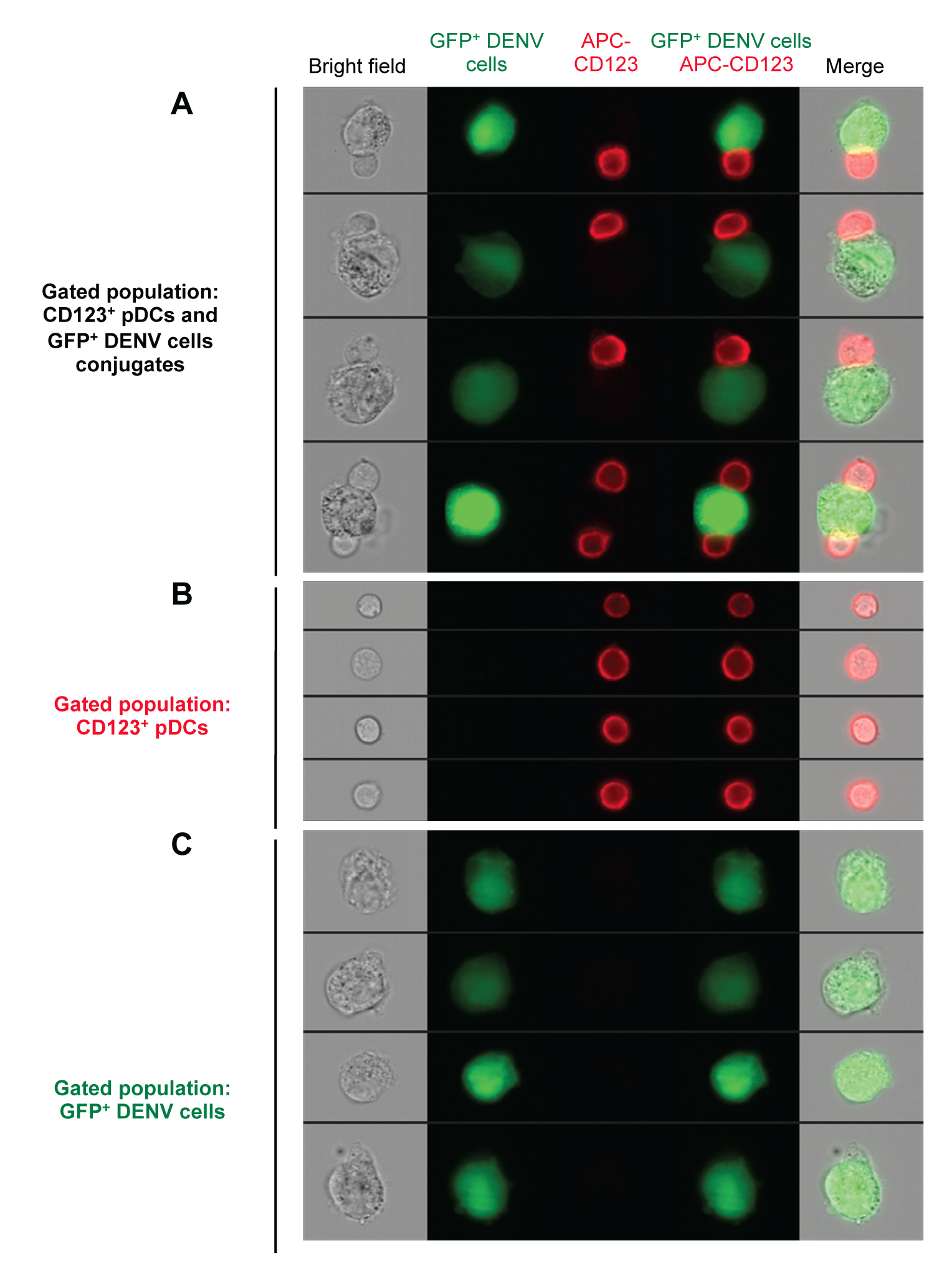

Supplement: Figure S13 — Analysis of the conjugates between pDCs and DENV infected cells by imaging flow cytometry analysis, related to Figure 8B . Imaging flow cytometry analysis (ImageStream) of DENV infected Huh7.5.1 cells, which stably express GFP, and co-cultured with pDCs for 8 hours, as described in the Figure 8B. pDCs are detected by the immunostaining of CD123, a pDC specific marker (APC-conjugated anti-CD123 antibody). Representative pictures of the cell population gated as conjugates between pDCs and GFP expressing DENV infected cells (A), of the cell population gated as pDCs, single cells (CD123 positive cells) (B), and of the cell population gated as DENV infected cells,(GFP positive cells) (C). Panels, as displayed from the left to the right, Bright field; GFP field; APC field; GFP/APC field and Merge. (TIF) [file ppat.1004434.s013.tif]

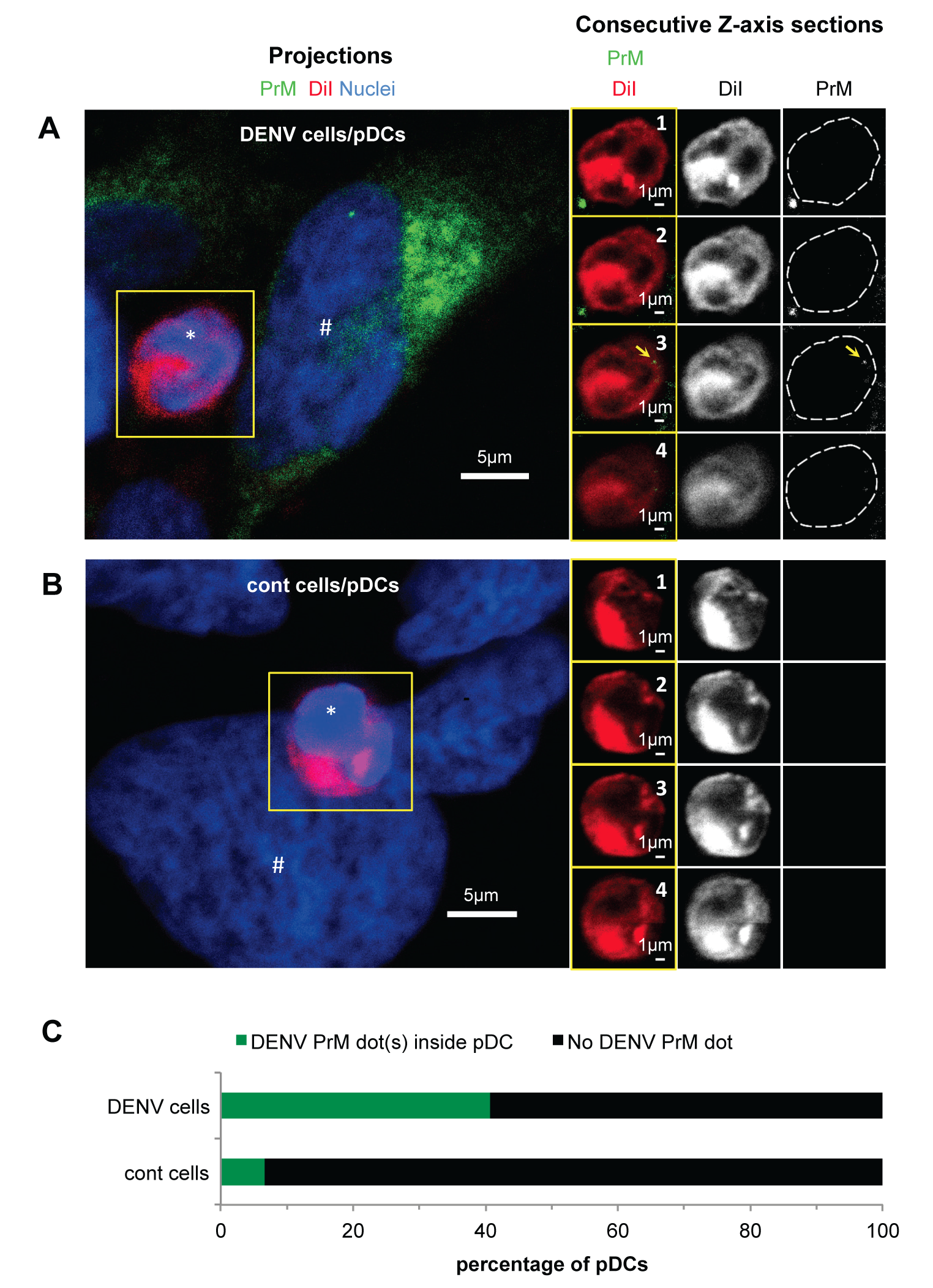

Supplement: Figure S14 — Transfer of DENV prM from DENV infected cells into co-cultured pDCs. Left panels, representative projections of confocal microscopy analysis of DENV prM (green) detected by immunostaining in co-cultures of DiI-stained pDCs (red) and DENV infected Huh7.5.1 cells (A), or uninfected (Cont) cells (B); nuclei (blue). Right panels, consecutive Z-axis sections with magnification of yellow-boxed pDC, shown in the corresponding projection. Cell contours on the DENV prM panels are labeled with dotted lines surrounding DiI staining. Yellow arrows; DENV prM protein dot inside pDC. (C) Results expressed as the percentages of DiI stained-pDCs containing prM protein dot(s). Similar results were obtained in 3 independent experiments, with ≈20 pDCs, surrounded by prM positive/DiI negative cell(s) observed per experimental condition. (TIF) [file ppat.1004434.s014.tif]
