# Supplementary material for: Sensing of Immature Particles Produced by Dengue Virus Infected Cells Induces an Antiviral Response by Plasmacytoid Dendritic Cells
Source: PLoS Pathog. 2014 Oct 23;10(10):e1004434. doi: 10.1371/journal.ppat.1004434 (PMC4207819; doi:10.1371/journal.ppat.1004434)
Supplement: Table S1 — Sequences of the primers for RT-qPCR and cloning. (DOCX) [file ppat.1004434.s015.docx]

**Table S1: Sequences of the primers for RT-qPCR and cloning**

| Cloning of DENV mutants and DENV surface proteins | |
| --- | --- |
| R88A/K90A/R91A for | CACAGAGCAGAAGCAGCATCAGTGGCACTCG |
| R88A/K90A/R91A rev | GCCACTGATGCTGCTTCTGCTCTGTGTTCTCCTGTG |
| ΔV51-L54 for | GTTCATGGCCCTGCGTTTCCTAACAATCCCACCAAC |
| ΔV51-L54 rev | GTTAGGAAACGCAGGGCCATGAACAGTTTTAATG |
| ADVprME_for | GATCCCCGGGACCGCCACCATGGTGAA |
| ADVprME_rev | GATCCCCGGGAGCTTGATATCAGGCCTGC |
| RT-qPCR | |
| hGAPDH 83U | AGGTGAAGGTCGGAGTCAACG |
| hGAPDH 287L | TGGAAGATGGTGATGGGATTTC |
| DENV-NSF | ACCTGGGAAGAGTGATGGTTATGG |
| DENV-NSR | ATGGTCTCTGGTATGGTGCTCTGG |
| Xef-1a 970L20 | CGACGTTGTCACCGGGCACG |
| Xef-1a 864U24 | ACCAGGCATGGTGGTTACCTTTGC |
| MxA-s | ACAGGACCATCGGAATCTTG |
| MxA-as | CCCTTCTTCAGGTGGAACAC |
| ISG15-s | GACAAATGCGACGAACCTCT |
| ISG15-as | CGGCCCTTGTTATTCCTCA |
